# Degradation of oxynitride based photoanodes

**DOI:** 10.1039/d5ta06368j

**Published:** 2025-12-15

**Authors:** Julian Hörndl, Jakub Zalesak, Franky E. Bedoya-Lora, Sophia Haussener, Simone Pokrant

**Affiliations:** a Department of Chemistry and Physics of Materials, Paris Lodron University Salzburg Jakob-Haringer-Str. 2A 5020 Salzburg Austria simone.pokrant@plus.ac.at; b Laboratory of Renewable Energy Science and Engineering, Ecole Polytechnique Federale de Lausanne 1015 Lausanne Switzerland

## Abstract

The production of green hydrogen *via* photoelectrochemical water splitting has the potential to play a vital part in the decarbonization of our energy economy. To commercialize this technology, the stability of the current photoelectrode materials needs to be improved. Therefore, it is essential to understand the processes causing degradation and define suitable figures of merit. This work investigates the degradation mechanisms of oxynitride electrodes focusing on TiO_2_ necked LaTiO_2_N particle-based photoanodes. Their degradation behaviour was assessed by chronoamperometries at 1.23 V *vs.* RHE in basic electrolyte. We identified two current decay processes based on a semi-empirical correlation between the measured chronoamperometries and a sum of two exponential decay terms. The time constant of the second exponential function is proposed as an alternative figure of merit for quantifying the stability of oxynitride based photoanodes. The applicability of this figure of merit to a wider range of oxynitride-based photoanodes and measurement conditions is demonstrated by evaluating previously reported chronoamperometries. By in depth analysis before and after chronoamperometry using STEM-EDX/EELS, HREM, ICP-MS, and XPS, we find experimental evidence that the performance decrease of the LaTiO_2_N photoanodes is caused by a combination of surface oxidation and cocatalyst dissolution.

## Abstract 2

Investigating the current evolution of oxynitride photoanodes over time revealed two exponential decay processes. In-depth analysis of the showcase LaTiO_2_N identified surface oxidation and cocatalyst dissolution as causes of the current decrease.

## Graphical abstract

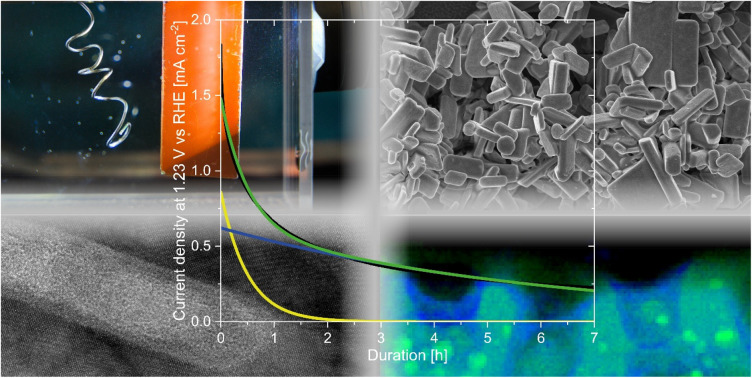

## 1 Introduction

The conversion of energy from renewable and CO_2_ free energy sources into chemical energy, such as solar fuels, has the potential to make a significant contribution to covering our future energy needs.^1–4^ Due to its lean setup, in comparison to the combination of a photovoltaic cell coupled to an electrolyser, photoelectrochemical water splitting (PEC) could provide an economically viable way for the generation of green hydrogen.^5–8^ A PEC system typically consists of two ohmically connected electrodes, where the photocathode provides the reaction sites for the hydrogen evolution reaction (HER) and the photoanode for the oxygen evolution reaction (OER).^5,9^

For this technology to be developed and commercialized, *i.e.* to be economically viable, the PEC system needs to fulfil four key requirements: efficiency, stability, scalability and sustainability.^2,6^ Concerning the efficiency of a PEC system, the generally accepted performance criterion is the solar to hydrogen efficiency (STH).^5,9^ To assess the minimum requirements for economic viability regarding these four key parameters, several technoeconomic studies have already been performed.^3,6,10^ In a recent publication Segev *et al.* reported that an economically competitive PEC system needs to achieve an STH of at least 10%, a lifetime of at least 10 years and manufacturing costs below $ 300 per m^2^ as criteria for scalabiltiy.^3^ In addition, live cycle assessments have been performed to determine the minimum operating time of a PEC device required to be net energy positive.^11^ Assuming a primary energy demand of 2110 MJ m^−2^ for a PEC device, Zhai *et al.* were able to determine the minimum operating time to be net energy positive as a function of the STH of the device.^11^ While technoeconomic analyses and live cycle assessments helped to assess the practicability and scalability of PEC systems,^1–3,6,11–13^ experimental research has mainly focused on increasing the efficiency of photoelectrodes.^5,14–16^ The required material properties of photocatalysts or photo absorbers in combination with cocatalysts, their morphological tuning on several length scales, and the establishment of adequate connectivity to a conductive substrate are well investigated and understood.^1,10,16,17^ It is generally agreed that the required efficiency level for economic viability has been reached in PEC, although further improvements are still desirable.^3,18^ However, since it is the combination of efficiency, stability and scalability that defines economic viability of PEC systems *versus* other technologies, research on stability phenomena should receive attention equal to that on efficiency.^8,16,17^

The first step to enhance photoanode stability is to identify the key degradation processes that are contributing to the observed performance decrease followed by the implementation of adequate mitigation strategies. In PEC, degradation can occur *via* charge-transfer, chemical decomposition, mechanical failure, or a combination of these three processes.^5,16,19^ Charge related degradation refers to the oxidation or reduction of the active material by (photogenerated) charge carriers.^19,20^ Chemical degradation describes processes taking place at the electrode/electrolyte interface without any light irradiation or electrical bias (*e.g.* chemical dissolution).^19,21^ In this context, the scientific community has renewed its interest in the subject of semiconductor electrode stability, which was firstly explored by Gerischer *et al.*^22^ More recently, the relevant physical and chemical processes contributing to photoelectrode degradation have been identified and reported for some material systems.^8,19,23,24^

A recent mechanistic study on the degradation behaviour of metal oxide photoelectrodes by Toma *et al.*^25^ revealed that the degradation of BiVO_4_ occurs *via* dissolution of the particle film upon exposure to the aqueous electrolyte. Similar dissolution-based degradation mechanisms were also observed by Knöppel *et al.* and Benavente Llorente *et al.* for WO_3_ and Fe_2_O_3_ based photoanodes.^26,27^ In all cases the dissolution rate of the photoanode material can be decreased by adjusting electrolyte composition and pH.^20,26–28^ In the case of oxynitrides such as TaON, some reports have suggested that TaON undergoes self-oxidation and that degradation seems to be caused by cocatalyst dissolution/redeposition.^29–31^ In addition a study on a thin film model system, using strongly nitrogen deficient LaTiO_2_N with photocurrent densities below 10 µA cm^−2^, reported surface oxidation after photoelectrochemical water splitting.^32^ Meanwhile for nitrides such as Ta_3_N_5_ the observed performance decrease is predominately caused by the formation of a thin amorphous TaO_*x*_N_*y*_ layer at the surface, which impedes the charge transfer across the interface.^33–36^ To inhibit this oxidation process, the application of a protective layer (*e.g.* GaN) and the addition of a cocatalyst such as CoP_i_ or Co_3_O_4_ have already been tested with some success.^33,35,36^ These examples demonstrate that the predominant degradation mechanism varies significantly not only with the type of photoactive material, but also with the entire photoelectrode system, *i.e.* the combination photocatalyst/cocatalysts and protective layers.

While most studies emphasize the need for consistent operating conditions to benchmark photoelectrode stability, the choice of metrics and protocols has often been tailored to the main degradation mechanism.^8,16^ This limits their applicability to systems where similar mechanisms dominate. One example is the degradation of WO_3_, where dissolution has been found to be predominant.^26^ In this context, the stability number (*S*), which describes the ratio of transferred electrons to the amount of dissolved catalyst, was identified as a suitable metric by Knöppel *et al.*^26,37^ Beyond that, metrics that do not rely on the exact degradation process were also proposed, although they suffered from different short comings: Werner *et al.* proposed current retention (Ret_CA_) extracted from long-term chronoamperometry measurements (≥6 h) under AM 1.5G illumination at 1.23 V *vs.* RHE (see eqn (1)).^16^1
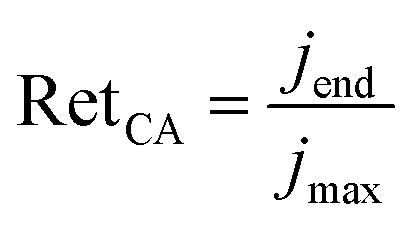
where *j*_end_ is the photocurrent density at the end of the degradation experiment and *j*_max_ is the maximum photocurrent density. The challenges of using Ret_CA_ as a figure of merit for photoanode stability lie in defining a suitable testing duration that is representative of the photocurrent decay and in identifying the maximum photocurrent density. The highest current density is typically observed at *t* = 0 s, but potential non-photocurrent related contributions, make it unreliable as *j*_max_.^38^ Assuming that additionally contributing processes decay notably faster than the photogenerated current, *j*_max_ can alternatively be approximated by an experimental photocurrent measured after a certain operation time (*e.g.* 10 min).^39^ Further the point in time for *j*_end_ needs to be standardized, as longer operation times automatically lead to lower values for *j*_end_ and Ret_CA_. Alternatively, the stability of photoelectrodes can also be assessed by performing linear sweep voltammetry before and after chronoamperometry, followed by comparing the measured photocurrent densities at 1.23 V *vs.* RHE before (*j*_1.23V_before__) and afterwards (*j*_1.23V_after__) (eqn (2)).^8,40^2
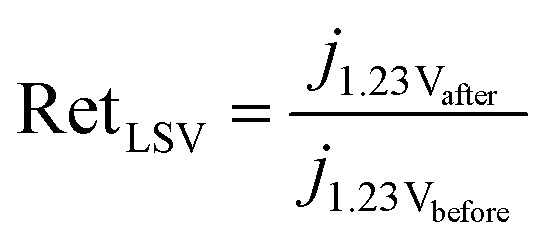


This figure of merit also has the drawback that meaningful stability comparisons require standardized chronoamperometry durations.

These examples highlight the challenge of developing general stability metrics or identifying general degradation processes that are applicable to most photoelectrodes. The lack of standardized protocols for assessing stability has likely contributed to researchers prioritizing efficiency over stability in the PEC field.^8,16^ In addition, photoelectrode degradation processes vary from electrode to electrode as a function of the employed material combinations, the exact electrode architecture and the operating conditions such as electrolyte, pH and applied bias.^8,16^ Therefore, a comprehensive understanding of degradation across different types of materials is essential for establishing relevant metrics and fabricating stable photoelectrode systems, which will eventually lead to economic viability.^14,23^ So far, previous reports have focused on degradation of oxides and nitrides,^25–28,33–36^ while oxynitrides have not been studied intensively, even though they have been employed successfully as photoanodes for catalysing the OER. One of the most studied oxynitrides is LaTiO_2_N (LTON), it exhibits competitive performance in both photoelectrochemical (between 2.5 and 8.9 mA cm^−2^ at 1.23 V *vs.* RHE^41–43^) and photocatalytic water splitting (apparent quantum yield of 65% at 420 nm^44^).

In this study we investigate the degradation mechanisms of oxynitride electrodes focusing on LTON particle-based photoanodes. Their degradation behaviour was assessed by comparing chronoamperometries at 1.23 V *vs.* RHE in basic electrolyte, with and without illumination. Further, gas chromatography was used to determine their faradaic efficiency. In all relevant cases, the photoanodes were investigated after the chronoamperometries using XRD, SEM, UV-Vis, STEM-EDX/EELS, HREM, and XPS, to analyse potential structural morphological or optical changes with respect to the pristine photoanodes. To examine potential cation dissolution from the photoanodes, the electrolyte was analysed using ICP-MS before and after the chronoamperometries. Based on our results we propose an alternative figure of merit for assessing the stability of oxynitride photoanodes. The applicability of this figure of merit to a wider range of oxynitrides and measurement conditions is demonstrated by evaluating previously reported chronoamperometries of oxynitride-based photoanodes.

## 2 Experimental

### 2.1 Material synthesis

The synthesis follows the procedure as described in ref. 45. To synthesize La_2_Ti_2_O_7_ (LTO) 6.25 mmol La_2_O_3_ (Sigma-Aldrich, 99.99%), 12.5 mmol TiO_2_ (Aldrich, >99%, Anatase) and 1.25 mol NaCl (VWR, 99%) were mixed with isopropanol and zirconia balls and placed on a roll mill for 3 h. Then, the precursor powder was washed with isopropanol and dried for 12 h at 70 °C. The dry powder was heated first at a rate of 10 K min^−1^ to 500 °C and then at a heating rate of 1 K min^−1^ to 1200 °C. The powder was held at this temperature for 10 h and subsequently cooled back to room temperature. Then the product was washed with 5 L of deionized water and afterwards dried for 10 h at 100 °C. To convert LTO to LTON, the obtained LTO powder was thermally treated for 18 h at 950 °C under 0.2 L min^−1^ NH_3_ flow before cooling back to room temperature under NH_3_ flow.

### 2.2 Electrode fabrication

The electrode fabrication is based on the process reported by Landsmann *et al.*^46^ Fluorine-doped tin oxide (FTO) substrates (2.5 × 1.25 cm, Solaronix, TCO15A) were cleaned by ultrasonication in 2% Hellmanex aqueous solution, water, and acetone. These FTO substrates were stored in isopropanol (Sigma Aldrich, p.a.). 43.5 mg LTON and 12.5 mg iodine were dispersed in 62 mL of acetone (Sigma Aldrich, p.a.) *via* ultrasonication for 10 min. After a waiting time of at least 1 h the dispersion was ultrasonicated for 10 min again. For the particle deposition, two FTO substrates were placed in the dispersion separated by a polytetrafluorethylene-bar with a thickness of 6.5 mm. A potential of 20 V was applied between the two substrates for 3.5 min, and the dispersion was stirred at 450 rpm for 10 s each minute. This process was repeated three times. After each deposition the dispersion was ultrasonicated for 2 min. In total eight photoanodes can be fabricated with one dispersion. Then, four post deposition steps were performed similar to Landsman *et al.*^41^ First, a necking procedure is performed as follows, the electrodes were dipped into an 0.2 M ethanolic TiCl_4_ solution for 10 s, dried for 1 h under ambient conditions and annealed for 30 min at 370 °C under 0.1 L min^−1^ NH_3_ flow. These samples will be referred as “LTON_bare” For the next step, the electrodes were dipped into a 0.2 M ethanolic TaCl_5_-solution for 10 s, dried for 1 h at ambient conditions and annealed for 30 min at 370 °C under 0.1 L min^−1^ NH_3_ flow. For co-catalyst deposition, the electrodes were dipped into a 0.05 M Ni(NO_3_)_2_ and a 0.05 M Co(NO_3_)_2_ ethanol solution for 20 s, dried for 1 h under ambient conditions and annealed in air for 10 min at 200 °C and at 150 °C respectively. These samples will be called “LTON_cocats”.

### 2.3 X-ray diffraction

X-ray diffraction (XRD) was performed using a Bruker D8 Advance diffractometer. This diffractometer with a goniometer radius of 280 mm is equipped with a fast solid-state LynxEye detector and an automatic sample changer. For the measurements thin zero-background single-crystal silicon sample holders are used. The LTO and LTON powders were drop coated onto the sample holder after mixing it with isopropanol. The LTON_cocats photoanodes, pristine and after stability testing, were placed directly onto the sample holder. As reference a bare FTO substrate was measured as well. The measurements were carried out in the 2*θ* range from 5° to 95° with a step size of 0.02° using a Cu Kα 1,2 radiation source. The resulting diffractograms were then compared with the corresponding references from the Inorganic Crystal Structure Database to ensure phase purity of the synthesized powders.

### 2.4 Scanning electron microscopy

The morphology of the LTON powder and the LTON based photoanodes was characterized with a Zeiss Ultra Plus 55 scanning electron microscope (SEM) using an in-lens secondary electron detector. For imaging an accelerating voltage of 5 kV was used. The working distance during the measurements was 4 mm. SEM images with magnifications of 25 000, 50 000 and 200 000 were acquired. Before the measurement the powders and photoanodes were sputtered with gold for 120 s.

### 2.5 Transmission electron microscopy

Transmission electron microscopy (TEM) lamellae were prepared using a Thermo Fisher Scientific Helios 5 FX dual beam microscope. Areas of interest were firstly covered with electron assisted C-deposition and further ion assisted C-deposition layers. Chunks of LTON material were cut along the long axes of the LTON particles and subsequently transferred to carbon grids. The thinning process was performed at accelerating voltages 30–2 kV and beam currents ranging from 2 nA to 25 pA.

TEM was performed with a JEOL-JEM-F200 TEM equipped with a cold field emission gun. The TEM was set to an acceleration voltage of 200 kV in both TEM and scanning TEM (STEM) mode. Selected area diffraction (SAED) was used to investigate the crystallinity of the particles and the orientation of the different facets for LTON powder. For this purpose, sample powder dispersed in ethanol was drop casted on a Cu-sample grid. The simulation of diffraction patterns was carried out using the JEMS software. STEM energy-dispersive X-ray spectroscopy (EDX) and electron energy loss spectroscopy (EELS) elemental mapping was performed using a Thermo Fisher Scientific Titan Themis equipped with a Super-X EDX detector system and a Gatan Quantum EELS spectrometer. The microscope was operated at an accelerating voltage of 300 kV and a beam current of ≈0.7 nA. The EELS data were acquired in dual-range mode using 5 mm entrance aperture, collection semi-angle of 100 mrad and dispersion of 0.5 eV per Ch. The recorded data were further processed using a Gatan Digital Micrograph 3 software.

### 2.6 X-ray photoelectron spectroscopy

The XPS measurements on LTON_bare photoanodes were performed with a PHI 5800 from Physical Electronics using monochromatic Al Kα (1486.6 eV) radiation with a power of 400 W. For signal detection an analyser angle of 90° was used. The surface of the LTON_cocats photoanodes was characterised by XPS (Axis Supra, Kratos Analytical). This system incorporates a monochromated Al K-alpha X-ray source (*hv* = 1486.69 eV). The pass energy of the analyser was set to 160 and 40 eV with step sizes of 1 and 0.15 eV for survey and high-resolution acquisition, respectively. CasaXPS software was used for the fitting and deconvolution of peaks.

### 2.7 UV-Vis spectroscopy

Diffuse reflectance and transmission measurements were carried out with a UV-Vis-NIR spectrophotometer (PerkinElmer, Lambda 1050). The transmission spectra of the photoanodes were measured in the wavelength range from 700 nm to 400 nm using 2 nm steps, and a bare FTO substrate as reference. The diffuse reflectance measurements were conducted with an integrating sphere in the wavelength range from 700 nm to 400 nm with 2 nm steps. The LTON powder was placed in a quartz cuvette, while photoelectrodes were directly placed in front of the pinhole of the integrating sphere. The corresponding backgrounds were determined using Ba_2_SO_4_ and a bare substrate respectively. Due to the opaqueness of the LTON based photoanode, the absorption coefficient *α* was approximated by the Kubelka Munk function, as calculated from the measured diffuse reflectance *R* (eqn (3)). For the determination of the band gap (*αhν*)^1/*n*^ is plotted as function of *hν* using the Tauc plot method. Since LTON has a direct band gap, *n* is 0.5.^47,48^ According to previous reports this method has an error of about ±0.05 eV.^48,49^3
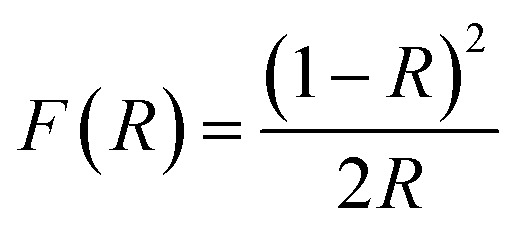


### 2.8 Induced coupled plasma mass spectroscopy

The concentration of La, Ti, Ta, Co and Ni in the electrolyte were measured with an Agilent 8900 Triple Quadrupole ICP-MS. Electrolyte samples of 10 mL were retrieved before and after 7 hours of continuous photo electrolysis and diluted ten times in 1% HNO_3_ to dissolve possible precipitates. The ICP-MS analysis was performed in helium mode. From the given volume *V* of the electrolyte (10 mL), the illuminated area exposed to the electrolyte A (0.125 cm^−2^) and the duration of the measurement *t* (7 h), average dissolution rates were calculated for the different cations based on the measured concentrations *c*_*x*_ in solution and their molar mass *M*_*x*_ (eqn (4)).^28^4
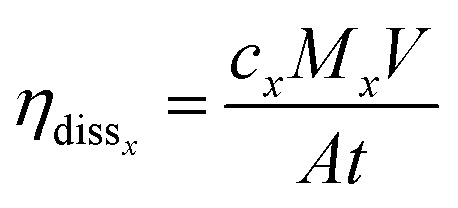
With respect to Ni cations the detected concentrations in the blanks were higher than the concentration detected after the chronoamperometry. Further investigations revealed that the high concentration of Ni in the blank was caused by insufficient purity of the used Na_2_SO_4_ and NaOH for the fabrication of the electrolyte. Due to the contamination of the pristine electrolyte, it was not possible to draw any conclusion regarding potential dissolution of Ni ions during the chronoamperometry.

### 2.9 Photoelectrochemical measurements

A 300 W Xenon lamp (Quantum Desing) with a light intensity of 1000 W m^−2^ and an AM 1.5G filter was employed as light source. In some cases, reduced illumination intensities of 48 kW m^−2^ and 120 kW m^−2^ were used. The measurements were performed using a three-electrode setup, incorporating a platinum wire as the counter electrode, an Ag/AgCl as the reference electrode (Metrohm, 0.21 V *vs.* SHE) and LTON-based photoanodes as the working electrode. A 0.1 M Na_2_SO_4_ (99%, Alpha Aesar) solution, with its pH adjusted to 13.4 by adding NaOH (>99%, Carl Roth), served as the electrolyte. Voltametric analysis was performed on the electrodes by sweeping the voltage from 0 V *vs.* RHE to 1.3 V *vs.* RHE, using a scan rate of 10 mV s^−1^, with and without illumination. The photocurrent was calculated by subtracting the current obtained in the dark from the current measured under illumination at 1.23 V *vs.* RHE.

To study the degradation of the LTON photoanodes under working conditions, chronoamperometric measurements were conducted at 1.23 V *vs.* RHE for seven hours, with and without illumination. The stability of the photoanodes was assessed by calculating the current retention after seven hours choosing the current values after 10 min or 1 h as *j*_max_ (eqn (1)).

### 2.10 Electrochemical impedance spectroscopy

Electrochemical impedance spectroscopy (EIS) was conducted under illumination at 1.2 V *vs.* RHE, using a 10 mV AC amplitude and a frequency range of 0.2–100 kHz. Measurements were taken on pristine LTON_bare and LTON_cocats photoanodes, as well as after each hour of chronoamperometry.

### 2.11 Faradaic efficiency measurements

Faradaic efficiencies were measured in a closed, partially electrolyte-filled chamber with an argon atmosphere, similar to Dilger *et al.*^50^ Before the measurement, the electrolyte was degassed by evacuation of the chamber and subsequent purging with Ar. A Keithly Source Meter 2601 was used as power source, which applied a constant voltage and recorded the current as a function of time. The gas composition inside the chamber was measured with an Inficon micro-GC fusion with the ability to detect molecular hydrogen, oxygen and nitrogen.

To account for potential crossover reactions, water electrolysis was used for calibration of the system. A potential of 2.1 V was applied between two platinum wires immersed in 200 mL 0.5 M H_2_SO_4_ for five hours and the molar fractions of hydrogen and oxygen were measured every 30 minutes. Using the volume of the chamber (*V* = 0.000406 m^3^), the temperature (*T* = 295.15 K) and the pressure inside the chamber (*p* = 120 000 Pa), and assuming ideality, the total amount of gas inside the chamber *n*_tot_ was calculated using eqn (5). With the total amount of gas inside the chamber *n*_tot_ = 0.01985 ± 0.00071 mol and the measured molar fractions of hydrogen and oxygen, *x*_H_2__ and *x*_O_2__ the evolved amounts of hydrogen and oxygen *n*_H_2_,exp_ and *n*_O_2_,exp_ were calculated using eqn (6).5
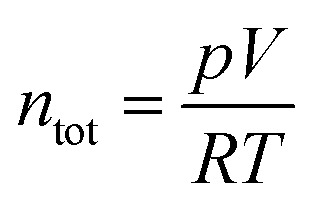

6 *n*_H_2_/O_2_,exp_ = *n*_tot_*x*_H_2_/O_2__

The expected amounts of hydrogen and oxygen based on the charge *Q*, *n*_H_2_,q_ and *n*_O_2_,q_ were calculated using Faraday's law (eqn (7)) with *z* = 2 for hydrogen and *z* = 4 for oxygen; *F* = 96 485 C mol^−1^. The faradaic efficiencies for the HER and the OER were then obtained from the ratios between the expected and the measured gas concentrations. To make sure the system was in equilibrium the zero point was set after the first hour of the measurement.7
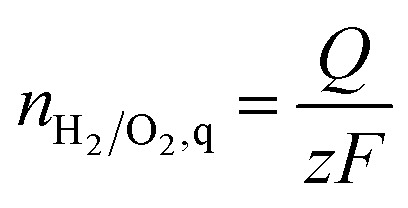


Averaged over three electrolysis measurements faradaic efficiencies of 0.994 ± 0.006 and 0.915 ± 0.006 were determined for the HER and the OER respectively. For the following measurements these values were used as calibration factors for determining the faradaic efficiencies of the HER and the OER, cal_HER_ and cal_OER_.

For faradaic efficiency measurements a constant potential of 1.23 V was applied between the LTON photoanode and a platinum wire for 5 hours and hydrogen and oxygen molar fractions inside the chamber were measured every 30 minutes. The LTON photoanode was illuminated using a 300 W Xenon lamp with a light intensity of 1000 W m^−2^ and an AM 1.5G filter immersed in 400 mL 0.1 M Na_2_SO_4_ (pH 13.4). The measured and expected amounts of hydrogen and oxygen were calculated using eqn (7)–(9). The faradaic efficiencies for the HER and the OER were then obtained by the ratios between the expected and the measured amounts of hydrogen and oxygen considering the corresponding calibration factors (eqn (8)).8
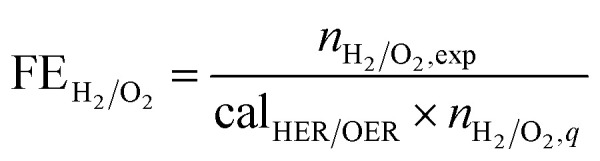


## 3 Results and discussion

### 3.1 LTON photoanodes

**Fig. 1:**
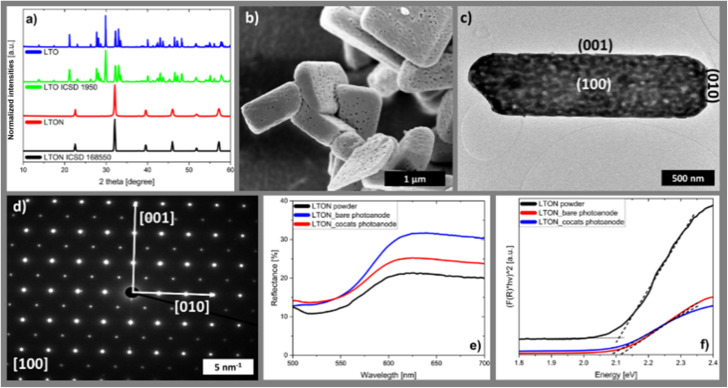
(a) XRD patterns of the synthesized LTO powder and the LTO ICSD 1950^54^ as well as LTON powder and LTON ICSD 168550,^51^ (b) SEM image of LTON powder (scale bar equals 1 µm), (c) HR-TEM (scale bar: 500 nm) and (d) SAED pattern of LTON powder (scale bar: 5 nm^−1^), (e) diffuse reflectance spectra and (f) Tauc-plot of the Kubelka–Munk function of LTON powder, LTON_bare photoanodes and LTON_cocats photoanodes. Linear extrapolation (dotted lines) to obtain the band gap.

Similar to other perovskite-related oxynitrides, LTON is synthesized by thermal ammonolysis of a layered precursor oxide, in this case LTO, at 950–1000 °C under constant NH_3_ flow for 18 hours.^45,51,52^ During this process nitrogen is incorporated into the LTO crystal lattice, shifting the valence band edge to a higher energy level, thus reducing the band gap. Consequently, LTON is able to absorb green and blue visible light.^52,53^ This is demonstrated by the fact that LTO is a white powder which turns red brown upon the transformation to LTON (Fig. S1a). Both the precursor LTO and LTON were investigated by XRD to verify their phase purity. When comparing the obtained diffractograms of LTO and LTON to the corresponding references from the ICSD (Fig. 1a),^51,54^ both show very good agreement. This indicates that both powders are single phase materials. LTON crystalizes in the space group *Imma*, which belongs to the orthorhombic crystal system, as reported previously. In this perovskite-type crystal lattice La^3+^ is located at the A-site and Ti^4+^ located at the B-site, while X sites are occupied by O and N, respectively.^55,56^ From an experimental point of view there is no indication for nitrogen ordering in the LTON lattice based on our measurements, while density functional theory calculations performed by Ninova *et al.* predict *cis* ordering in the bulk and *trans* ordering at the particle surface.^53,57,58^

The particle morphology investigation by SEM confirmed that LTON consists of brick shaped platelets with their sizes ranging from 400 nm to 3.3 µm and their thicknesses ranging from 150 to 700 nm measured over 50 particles (Fig. 1b). At the surface of these particles 10–25 nm sized pores are visible. These pores belong to a network of cavities inside the LTON particle.^59,60^ This network is the origin of the contrast variations observed in the TEM image (Fig. 1c). Due to this network the synthesized LTON powders have enhanced surface areas between 10 and 15 m^2^ g^−1^ (Fig. S2).

The crystallinity of LTON on the particle level was investigated by selected area (electron) diffraction (SAED). The diffraction patterns were acquired from isolated particles on the grid (representative particle Fig. 1c), oriented with the largest surface perpendicular to the beam. They show a regular and rectangular distribution of the diffraction spots (Fig. 1d) consistent with the crystal structure as determined by XRD. This indicates that the investigated particle is monocrystalline with a platelet-like shape.^56^ Electron diffraction simulations identify the viewing direction of the pattern as the [100] direction. This means that the largest surfaces of the particle belong to the {100} plane family, while the smaller side facets consist of {001} and {010} planes. The same observations have already been reported by previous studies on LTON particles.^45,59^

**Fig. 2:**
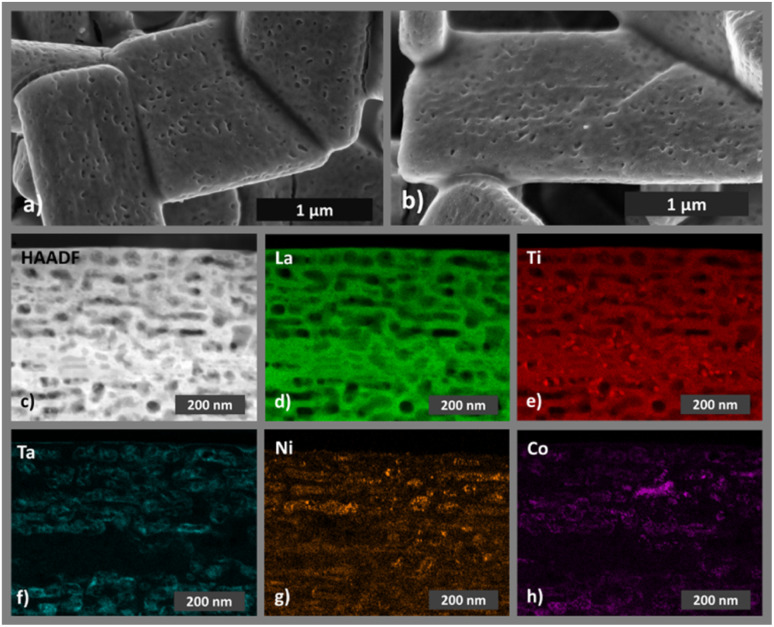
Representative SEM images of (a) a pristine LTON_cocats photoanode and (b) a LTON_cocats photoanode after 7 h chronoamperometry under illumination. (c) HAADF STEM image of a lamella extracted from a pristine LTON_cocats photoanode. STEM-EDX elemental maps of the same lamella for (d) lanthanum, (e) titanium, (f) tantalum, (g) nickel and (h) cobalt.

The optical properties of the LTON powders were assessed by diffuse reflectance spectroscopy (Fig. 1e). In the reflection data an edge is visible at around 600 nm. Since LTON is known to exhibit a direct band gap,^47,48^ the Tauc plot (eqn (3)) of the Kubelka–Munk function (Fig. 1f) for direct band gap materials was used to determine the band gap of the LTON powder (2.11 ± 0.05 eV) by extrapolation (Fig. 1f and Table S1). The obtained value is in good agreement with previously reported bad gaps of LTON powders^45,49,59^ and is considered as advantageous for visible light driven water splitting.^5,10^ The LTON-based photoanodes (Fig. S1b) prepared by electrophoretic deposition are composed of 4.5 to 6 µm thick films of irregularly stacked LTON particles on top of conductive fluorine-doped tin oxide (FTO) substrates, as observed by SEM (Fig. 2a and S3) and by profilometry (Fig. S4). After LTON powder deposition, TiO_2_ necking is performed by dipping into the ethanolic TiCl_4_ solution followed by annealing under NH_3_ flow similar to Nishimura *et al.*^61^ We will refer to photoanodes with TiO_2_ necking and no further processing as LTON_bare. We attribute the matrix-like structure, which connects and partly covers the particles, to TiO_2_ necking, because this structure is visible on the LTON photoanodes before and after cocatalyst deposition (Fig. S3). A similar morphology has been observed by Nishimura *et al.* as a result of TiO_2_ necking.^61^ It is well known that necking leads to the formation of TiO_2−*x*_ links between the LTON particles.^61,62^ Necking not only increases the mechanical stability of the photoanodes but also the charge transfer between the LTON particles, resulting in performance improvement of the photoanodes.^50,61^ Additional post deposition layers were applied to necked LTON photoanodes by dipping in TaCl_5_, Ni(NO_3_)_2_ and Co(NO_3_)_2_ solutions. We will refer to photoanodes with all four post modifications steps as LTON_cocats.

The XRD pattern of a representative LTON_cocats photoanode showed that the film consists of phase pure LTON comparable to the pristine powder (Fig. S5). The additional peaks in the diffraction pattern can be attributed to the FTO substrate. Furthermore, no morphological differences were detectable between pristine LTON powder particles and particles on LTON_bare and the LTON_cocats photoanodes by SEM (Fig. 1b and S3), suggesting that the post modification steps did not affect the LTON. XPS analysis confirmed the presence of Ta, Co and Ni on the surface of LTON_cocats electrodes (Fig. S6). To investigate the morphology and elemental distribution of LTON_cocats photoanodes, HAADF imaging and STEM-EDX elemental mapping was performed on a lamella extracted from an LTON_cocats photoanode particle. A porous network comparable to pristine LTON was observed within the LTON_cocats particle by HAADF imaging and La and Ti elemental mapping (Fig. 2c–e). The areas with higher intensities in the Ti map corelated to LTON pore locations. We assume that these pores were filled with TiO_2_ necking material. Furthermore, all cocatalysts (Ta, Ni and Co compounds) were detected on the LTON particle (Fig. 2f–h). Thereby, the distribution of Ta appeared to be quite homogenous consistent with a Ta_2_O_5_ layer, while Ni and Co were distributed more heterogeneously pointing to nanosized structures or particles. These results are in agreement with the literature where it is stated that dipping into ethanolic TaCl_5_ solution followed by annealing under NH_3_ flow led to the formation of a thin amorphous Ta_2_O_5_ layer,^41,63^ while by dipping into ethanolic Ni(NO_3_)_2_ and Co(NO_3_)_2_ solutions followed by annealing in air an amorphous NiO_*x*_ layer and crystalline CoO_*x*_ nanoparticles were formed.^41,50,64^ These observations confirm the successful fabrication of LTON based photoanodes with Ta, Ni and Co as cocatalysts.

In terms of the optical properties, we found through visual inspection that the particle films on top of the LTON_bare and LTON_cocats photoanodes exhibited the same reddish-brown colour (Fig. S1b) as the LTON powder and that they are opaque at a thickness of around 5 µm. This indicates that almost all incident light is either absorbed or reflected by the particle film. This result was confirmed by UV-Vis transmission spectroscopy, which showed that almost no transmitted light was detected across the entire visible light range for both LTON_bare and LTON_cocats photoanodes (Fig. S7).

Moreover, the diffuse reflectance spectra of both photoanode types were very similar regarding their absorption edge and intensity (Fig. 1e) compared to the powder. The direct band gap extrapolated from the Tauc-plot of the Kubelka–Munk function (Fig. 1f) was 2.09 ± 0.05 eV for LTON_cocats photoanodes and 2.11 ± 0.05 eV for LTON_bare photoanodes, which is almost identical to the value obtained for the direct band gap of the LTON powder (2.11 ± 0.05 eV). Thus, the optical properties of the LTON powder and the photoanode were found to be unchanged, after necking and cocatalyst deposition, within the precision of our measurement. In summary, neither the photoelectrode fabrication process nor the post-modifications steps significantly altered the pristine LTON powder with respect to its optical or morphological properties.

### 3.2 Performance and stability of oxynitride photoanodes

**Fig. 3:**
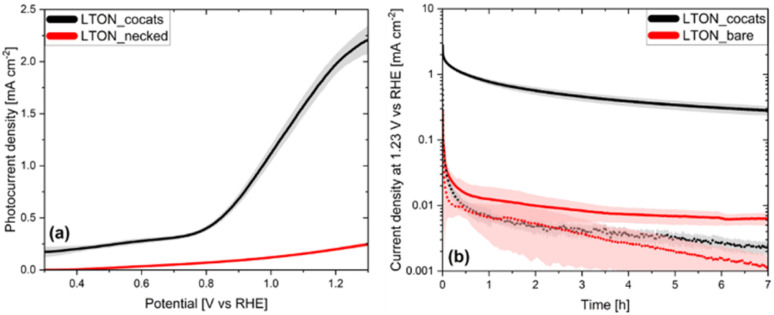
(a) Photocurrent densities as a function of the applied bias potential of the average over five LTON_bare photoanode and LTON_cocats photoanode (b) 7 h chronoamperometries of the average over three LTON_cocats photoanode and a LTON_bare photoanode at 1.23 V *vs.* RHE. The corresponding chronoamperometries without illumination are shown as dotted line.

In agreement with literature, the photocurrent density at 1.23 V *vs.* RHE was selected as an indirect measure for the performance of the LTON photoanodes.^3,16^ All measurements were carried out in 0.1 M Na_2_SO_4_ solution, with its pH adjusted to 13. The photogenerated current of bare FTO substrates was <1 µA cm^−2^ at 1.23 V *vs.* RHE (Fig. S8a). After the deposition of LTON and TiO_2_ necking, a mean photocurrent density of 203 ± 20 µA cm^−2^ was found at 1.23 V *vs.* RHE averaged over five LTON bare photoanodes (Fig. 3a and S9a). As the photocurrent density of the bare FTO was negligible, the observed photocurrent of these electrodes was entirely generated by LTON. In the next step we investigated the influence of the three cocatalyst systems on the photocurrent density (see Fig. S8a). The addition of only CoO_*x*_, NiO_*x*_ or Ta_2_O_5_, respectively, resulted in sevenfold (1.44 mA cm^−2^), fivefold (1.08 mA cm^−2^) and negligible photocurrent density increase (0.22 mA cm^−2^) at 1.23 V *vs.* RHE. Combining CoO_*x*_ with either Ta_2_O_5_ or NiO_*x*_, photocurrent densities of 1.77 and 1.99 mA cm^−2^ at 1.23 V *vs.* RHE were obtained. This shows that the performance of the LTON based photoanodes is significantly increased by both NiO_*x*_ and CoO_*x*_, while the Ta_2_O_5_ layer has only a small influence. Due to its high thermal and chemical stability, this layer is thought to serve a protective function rather than to increase photocurrent density, as reported elsewhere.^41,46,63^ It has also been proposed that the NiO_*x*_ layer facilitates hole extraction and reduces photo corrosion by acting as hole storage material, while the CoO_*x*_ nanoparticles promote charge separation, resulting in decreased onset potentials and overall higher photocurrents, in agreement with our experimental observations.^41,46,50,63,64^ Applying all three cocatalyst systems (CoO_*x*_, Ta_2_O_5_, NiO_*x*_), the photocurrent density increased to 2.10 ± 0.10 mA cm^−2^ at 1.23 V *vs.* RHE for LTON_cocats, again averaged over five photoanodes (Fig. 3a and S9a). Hence their performance was ten times higher than that of LTON_bare photoanodes, but still noticeably lower than some of the previously reported LTON based photoanodes.^43,65^ This is due to the use of an electrode fabrication method *via* electrophoretic deposition favouring scalability over performance.^46^ Focussing only on LTON based photoanodes fabricated *via* electrophoretic deposition our photocurrent density of 2.10 ± 0.10 mA cm^−2^ is comparable to photocurrent densities reported in the literature.^41,42,46^ Altogether, LTON-based photoanodes are considered promising in terms of efficiency and, therefore, merit an in-depth stability investigation.^10,44,52^

To investigate their stability, chronoamperometric studies were performed on LTON_cocats and LTON_bare photoanodes as well as on bare FTO substrates at 1.23 V *vs.* RHE for seven hours (Fig. 3b, S8b and S9b) under illumination and in the dark. For the bare FTO substrates a constant current around 2 µA cm^−2^ was observed both under illumination and in the dark (Fig. S8b). In the case of LTON_cocats photoanodes under illumination the current density dropped from an initial value of almost 3 mA cm^−2^ to less than 1 mA cm^−2^ within the first 30 minutes. It further decreased at a slower rate to a final value of 0.28 mA cm^−2^ after seven hours. Concerning LTON_bare photoanodes, the initial current of 0.3 mA cm^−2^ fell within the first five minutes to 0.03 mA cm^−2^ before it stabilized at values around 0.007 mA cm^−2^ after the first hour. Regarding the current densities in the dark, no significant differences were observed between LTON_bare and LTON_cocats photoanodes. Both electrodes started with current densities around 0.3 mA cm^−2^ and stabilized, after a steep drop during the first 30 min, at values below 0.01 mA cm^−2^. Due to the wide range of time scales reported in the literature for chronoamperometric analyses, it is often difficult to compare the stability of different photoelectrode materials quantitatively. Upon a qualitative comparison with chronoamperometries reported for BiVO_4_, Fe_2_O_3_ and WO_3_, notable differences in the progressions of these curves are evident with respect to LTON (Fig. S10a–d), such as much more moderate current density decay.^20,25–28^ The observed differences in the photocurrent decay patterns suggest that LTON might have a different degradation mechanism than these oxides. Meanwhile the comparison with chronoamperometries from other oxynitrides (*e.g.* SrTaO_2_N) or nitrides (*e.g.* Ta_3_N_5_) revealed a similar curve progression (Fig. S10d–f), suggesting that the underlying degradation mechanism might be similar across (oxy)nitrides. This finding motivates a more detailed analysis of the chronoamperometric behaviour of (oxy)nitrides, with a particular focus on LTON.

To quantify the stability of the electrodes based on our chronoamperometric measurements, we calculated the current retentions according to eqn (1). When *j*_max_ was set to the current densities measured at *t* = 10 min and *j*_end_ to the value measured at *t* = 7 h, the current density retentions Ret_CA_ obtained for LTON_cocats and LTON_bare photoanodes were comparable with 20% and 23%, respectively. Repeating the calculation with *j*_max_ = *j*_(*t*=1h)_ and *j*_end_ = *j*_(*t*=7h)_, we obtained 35% current retention for LTON_cocats photoanodes and a 11% higher value (46%) for LTON_bare photoanodes, indicating that after one hour operation LTON_bare photoanodes were more stable than the LTON_cocats photoanodes. To evaluate whether the improved stability of LTON_bare photoanodes was related to their lower current densities, we conducted chronoamperometries at higher illumination intensities (48 and 120 suns). Thereby the current density increased five to ten times, resulting in final currents of 0.035 mA cm^−2^ and 0.07 mA cm^−2^, respectively (Fig. S11). Using the current densities measured after 1 h as *j*_max_, current retentions of 49% (48 suns) and 59% (120 suns) were obtained. Thus, we concluded that the higher stability of LTON_bare photoanodes was not related to their lower current densities. Nonetheless the long-term stability of both electrode types, LTON_bare and LTON_cocats, falls significantly short of meeting industrial stability requirements, that is in the order of years.^6,10^ Similar observations regarding the poor stability of based LTON photoanodes were also reported by Feng *et al.* and by Mao *et al.*^42,66^

To gain further insight into the degradation behaviour of (oxy)nitride photoanodes, the average current density decay of three LTON_cocats and three LTON_bare samples was analysed (Fig. 3b). At first sight the current density profiles plotted on a linear scale (Fig. S12) suggested an exponential decay. This exponential character can be rationalized by assuming that some of the interfaces act as capacitors that are charged at the beginning of the experiment, *i.e. via* light exposure and/or *via* the applied bias, as already suggested in the literature.^13,67^ Using the concept of a capacitor to model this process, the maximum generated current by this process is *I*_0,cp_
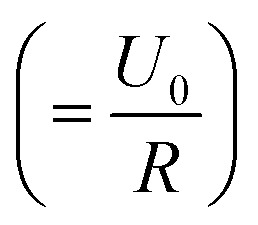
 and the characteristic decay time *τ* (*τ* = *RC*, with *R* being the resistance and *C* the capacitance), corresponds to the time at which the current has decreased to the *e*^−1^-fold of its initial value (approximately 36.8%).9
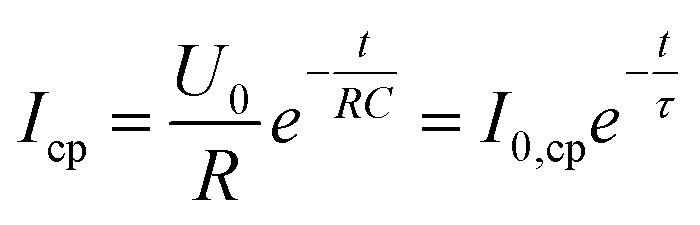
Thus, the measured chronoamperometries were fitted with an exponential decay function (eqn (9)). However, the correlation between the experimental LTON chronoamperometry and the fitted curve is not satisfying, neither for LTON_bare nor for LTON_cocats, as it is evident in Fig. S12a and c and reflected in the low coefficients of determination (*R*^2^ – values of 0.235 and 0.859).

Inspecting the experimental chronoamperometry curves of LTON_bare and LTON_cocats photoanodes on a logarithmic scale (Fig. S13) suggested that a second exponential decay, here labelled with the subscript “pc”, takes place at longer time scales, as indicated by the current retention calculations presented above. Both effects are accounted for in eqn (10).10
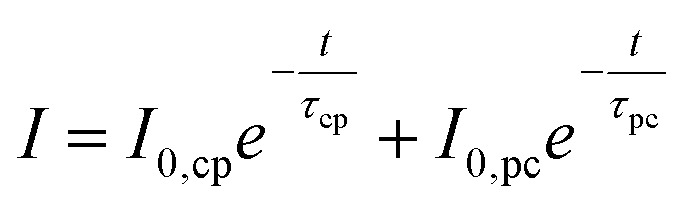


Using two exponential terms in the fitting function, an excellent representation of the experimental chronoamperometry was obtained for both LTON_bare and LTON_cocats photoanodes (Fig. S13b and d). This was further demonstrated by the notably higher *R*^2^ values of 0.935 and 0.994. Thus, we propose that, for an adequate description of the decay pattern of LTON_bare and LTON_cocats photoanodes, two exponential terms with different decay rates are necessary.

In the next step, we check whether our fitting models, which contain either one or two exponential decay functions, are applicable to previously reported oxynitride-based photoanodes.^31,42,66,68–70^ For this, eleven chronoamperometries from six different reports were fitted (Tables S2, S3 and Fig. S14–S24). The results are listed in Table 1. With respect to the fits using a single exponential term we find that more than 80% of the *R*^2^ values were below 0.9. Concerning the fits using two exponential terms, all *R*^2^ values were above 0.970, and more than half of the fitted curves were above 0.990. Visually, the two-exponential decay fits represent more accurately the experimental chronoamperometries compared to single-exponential fits (Fig. S14–S24). This is an indication that two current decay processes with two different time constants *τ*_cp_ and *τ*_pc_ occur in parallel for oxynitride-based photoanodes (eqn (10)). These results strengthen our own observations for LTON and extend their validity to several oxynitride based material systems. Therefore, we evaluate LTON based photoanodes with a fitting function based on two exponential decay functions.

**Table 1:** Fitting results for chronoamperometric measurements under illumination reported in the literature and in this work using a decay function consisting of two exponential terms.^31,42,66,68–70^ Photocatalysts with the label “dark” were measured without illumination

Photo-catalyst	Cocatalyst	*R* ^2^ one exponential	*R* ^2^ two exponentials	*τ*_cp_ [min]	*τ*_pc_ [min]	References
LaTiO_2_N	Co_3_O_4_	0.534	0.987	0.90	235	Feng *et al.*^42^
LaTiO_2_N	Co_3_O_4_	0.758	0.987	3.67	192	Mao *et al.*^66^
Na_0.1_La_0.9_TiO_2.2_N_0.8_	Co_3_O_4_	0.549	0.995	0.26	112	Mao *et al.*^66^
SrTaO_2_N	—	0.721	0.982	1.83	62.9	Zhong *et al.*^68^
SrTaO_2_N	CoP_i_	0.756	0.973	1.47	86.5	Zhong *et al.*^68^
BaNbO_2_N	FeO_*x*_ & Co(OH)_*x*_	0.828	0.991	2.43	86.6	Seo *et al.*^69^
TaON	—	0.986	0.999	0.85	37.7	Higashi *et al.*^31^
TaON	CoO_*x*_	0.117	1.000	0.42	122	Higashi *et al.*^31^
TaON	IrO_*x*_	0.772	0.988	2.87	130	Higashi *et al.*^31^
TaON	CoO_*x*_	0.104	1.000	0.29	153	Higashi *et al.*^31^
Ta_3_N_5_	—	0.956	0.999	4.59	89.2	He *et al.*^70^
LaTiO_2_N	CoO_*x*_ & NiO_*x*_	0.859	0.994	30.3	387	This work
LaTiO_2_N	—	0.235	0.935	4.02	335	This work
LaTiO_2_N_dark	CoO_*x*_ & NiO_*x*_	0.779	0.944	0.60	130	This work
LaTiO_2_N_dark	—	0.506	0.963	1.14	172	This work

Firstly, we focus on the contribution associated with the short characteristic time constant, *i.e. τ*_cp_, since it was typically more than 30 times shorter compared to *τ*_pc_ (Table 1). Thus, after a certain time (in most cases after 10 min and in all cases after 1 h) the contribution of the first exponential term, described by *τ*_cp_ and *I*_0,cp_, was reduced to less than 10% of the overall current and thus negligible (Table S3). *I*_0,cp_ describes the maximum contribution to the overall current of the first exponential term at *t* = 0 s, representing 31 to 97% of the measured current at that time (Table S4), *i.e.* the photocurrent density at *t* = 0 s has substantial contributions that decay in the minute range.

To investigate how the observed current decays with short time constants were connected to light exposure, additional chronoamperometries without illumination were performed on LTON_bare and LTON_cocats photoanodes (Fig. 3b) and their characteristic parameters were calculated (Table 2) by fitting with a two exponential decay function (eqn (10)). The current densities *I*_0,cp_ showed a weak illumination dependence (by factors 1.5 to 1.7), while the *I*_0,pc_ increased by a factor of 38 with light exposure in the case of LTON_cocats. LTON_bare showed comparable current density ratios for both *I*_0,cp_ and *I*_0,pc_, as it was to be expected from its weak performance as photoanode during linear sweep voltammetry. These results suggested that the first process is not light induced, whereas the second is. One exponential current decay process that is not light dependent is the discharge of a capacitor. The capacitance of a system is the ratio *Q*_cp_/*V*_tot_, with *Q*_cp_ = *I*_0,cp_*τ*_cp_ being the charge released from the capacitor and *V*_tot_ the sum of the applied bias and photo-induced bias (=open circuit voltage).^71^ For LTON_bare photoanodes, the calculated capacitance under illumination (16.3 mF) was three times the capacitance without illumination (5 mF) (Table S4). With capacitances of 958 mF under illumination and 16 mF in the dark, this difference was even more pronounced for the LTON_cocats photoanodes (Table S4). Analysing the current density decay for different combinations of cocatalyst decorations (Table S5), we find that the capacitance of the photoanodes increased with the addition of cocatalyst layers and the increase was significantly higher under illumination than in the dark. The observed increase of the capacitance under illumination is likely due to a light induced separation of charge carriers in the photoanode, as proposed by Peter*.*^67^ On the model system of hematite photoanodes the authors discussed that the sudden exposure to illumination lead to the separation of electrons and holes in the photoanode, with the former being driven to the bulk and the latter to its surface, thus, generating a fast-decaying current, that was not associated with charge carriers crossing the photoanode electrolyte interface. In analogy we propose that the part of the measured current density at *t* = 0 s related to *I*_0,cp_ in the oxynitride based photoanodes is caused by the capacitive behaviour of the photoanode. Therefore, the measured current *I*(*t* = 0 s) is not representative for the maximum photocurrent, since it contains large contributions not related to the oxidation of water.^9^

**Table 2:** Capacitive and photocurrent related contributions to the current density obtained for LTON_bare and LTON_cocats electrodes under illumination and in the dark obtained by fitting with two exponential terms

	Under illumination				No illumination			*τ*_pc_ [min]
	*I*_0,cp_ [mA cm^−2^]	*τ*_cp_ [min]	*I*_0,pc_ [mA cm^−2^]	*τ*_pc_ [min]	*I*_0,cp_ [mA cm^−2^]	*τ*_cp_ [min]	*I*_0,pc_ [mA cm^−2^]	
LTON_bare	0.11	4.02	0.013	355	0.09	1.1	0.011	172
LTON_cocats	0.86	30.3	0.61	387	0.56	0.6	0.016	130

Assuming that the first exponential decay term of eqn (10) arises from a capacitive behaviour, it is reasonable to attribute the current decay described by the second exponential term to electrode degradation. In this context, *I*_0,pc_ corresponds to the photocurrent density obtained at *t* = 0 s and *τ*_pc_ indicates the time constant of the decay. For the dark measurements at 1.23 V *vs.* RHE, *I*_0,pc_ has a value of 16 µA cm^−2^ for LTON_cocats indicating that, even without illumination, a small current with a decay time constant *τ*_pc_ is present (Table 2).

It is interesting to revisit one of the metrics for stability, *i.e.* photocurrent retention (eqn (1)), which is defined as the ratio between the photocurrent *j*_end_ at a given time *t* and *j*_max_, the photocurrent maximum. So far, either the measured current at *t* = 0 s or after an arbitrary period (*i.e.* 10 min or 1 h in this article) has been used as *j*_max_.^16,72^ However, at *t* = 0 s the capacitive current contribution is large, as shown in Table S4. Furthermore, the large variation of *τ*_cp_ for different systems (Table 1) indicates that the waiting time until the capacitive contribution has become negligible, varies substantially as a function of the investigated system. Thus, it is unlikely to find a universally applicable delay ensuring that the measured current corresponds mainly to the photocurrent. Therefore, *I*_0,pc_ is proposed as *j*_max_ for the calculation of photocurrent retention (Table S6). *I*_0,pc_ offers the advantage that capacitance-related contributions are not included. In addition, as an alternative to current retention, we propose *τ*_pc_ as a figure of merit to assess the stability of photoanodes. Longer characteristic time constants correspond to slower exponential decay, thus indicating better stabilities. In order to evaluate whether the proposed metrics for stability, *i.e.* current retention and characteristic time constant *τ*_pc_, are correlated, the obtained time constants *τ*_cp_ and *τ*_pc_ were plotted as function of the current retention calculated with *j*_max_ = *I*_0,pc_ and *j*_end_ = *j*_*t*=60min_ (Fig. S25). We find that *τ*_cp_ is not correlated to current retention, and thus electrode stability, while *τ*_pc_ and the current retention are correlated *via* an exponential term, shown as linear dependence in the log plot in Fig. S25. This analysis confirms that the fitting parameter *τ*_pc_ contains the same information as the current retention and qualifies thus as an alternative metric for stability. Hence, using *τ*_pc_ as a stability metric offers two key benefits. First, it is unaffected by the duration of the experiment, provided the chronoamperometry is long enough for accurate curve fitting. Second, it accounts for initial capacitive effects that might not related to degradation.

Using *τ*_pc_ as a metric for stability, we compared the effect of different cocatalyst layers on the stability of the LTON photoanodes obtained from the chronoamperometries displayed in Fig. S8b. The results are listed in Table S7. The cocatalyst systems CoO_*x*_ and NiO_*x*_ did not strongly affect the stability of the electrodes, although they increased the performance, as discussed above. The Ta_2_O_5_ layer, however, resulted in improved stability. For example, in the case of LTON_bare photoanodes decorated with CoO_*x*_ and NiO_*x*_ lead the addition of Ta_2_O_5_ to a *τ*_pc_ of 6.27 h instead of 3.92 h. These results confirm that Ta_2_O_5_ serves a protective function, as already suggested in the literature.^41,63^ Interestingly, although LTON_bare photoanodes did not contain any cocatalysts system to either decrease charge accumulation or protect the surface, it showed better stability than LTON_cocats (Table 2).

### 3.3 Investigation of degradation processes in LTON-based photoanodes

To improve LTON-photoanode stabilities, it is critical to identify potential degradation processes taking place during photoelectrochemical water splitting. Therefore, we discuss in the next sections our findings concerning degradation processes. First, we investigate potential changes in the optical properties followed by electrochemical experiments to locate the origin of the degradation.

Concerning the optical properties, we could not observe significant changes. The diffuse reflectance spectra of LTON_cocats and LTON_bare photoanodes after stability testing are almost identical to the diffuse reflectance spectra of the corresponding pristine photoanodes (Fig. S26a). With 2.11 ± 0.05 eV and 2.09 ± 0.05 eV also the direct band gaps of LTON-necked and LTON_cocats photoanodes did not change during the chronoamperometries (Fig. S26b and Table S1). The observed photoelectrochemical degradation does not seem to affect the optical properties of the LTON photoanodes.

**Fig. 4:**
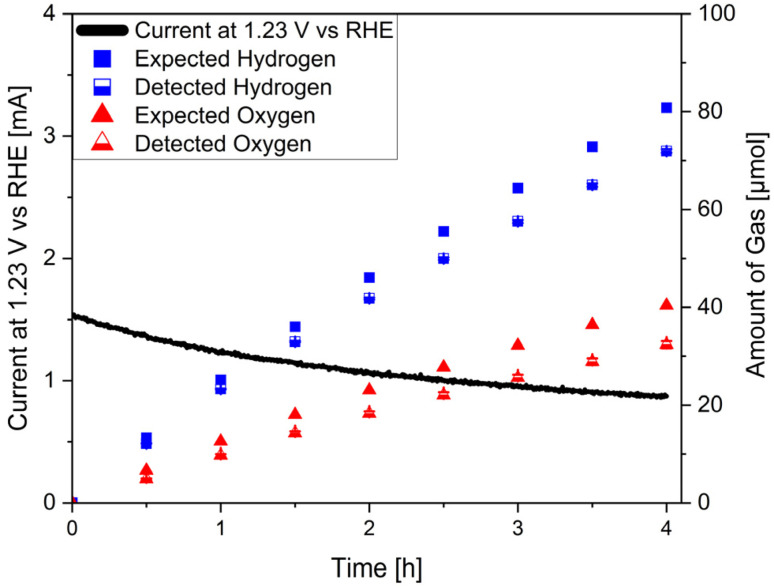
Faradaic efficiency measurements of LTON_cocats photoanodes under illumination.

To investigate possible charge-related degradation processes, faradaic efficiency measurements were performed for four hours in a two-electrode configuration under AM 1.5G illumination, at 1.23 V *vs.* RHE (Fig. 4). To ensure that the system was in equilibrium, the measurement was started after the first hour of operation. Averaged over four measurements, faradaic efficiencies of 88.2 ± 1.9% and 89.0 ± 3.3% were observed for the HER and the OER respectively. Thus, for both half reactions about 10% of measured charge did not contribute to the desired reaction. Based on the literature about the OER on oxynitrides, a likely side reaction is the oxidation of N^3−^ at the surface of the LTON particles.^16,32,62^ If 10% of the total charge (*ca*. 1.6 C) contributed to nitrogen evolution, this would lead to a total amount of 2.9 µmol. However, during the measurements only 0.85 ± 0.14 µmol of nitrogen were detected. When subtracting the nitrogen background (0.72 ± 0.09 µmol) in the chamber, less than 10% of the expected amount of nitrogen was detected. Therefore, it is unlikely that the root cause for the reduced faradaic efficiency was nitrogen evolution. Since the faradaic efficiency decrease was symmetric, *i.e.* comparable on the hydrogen and on the oxygen evolution side and considering the absence of a membrane in the setup, back reaction effects might occur *via* reduced/oxidized species diffusing to the anode/cathode. Although some nitrogen evolution might take place, we exclude charge related degradation as the dominant degradation mechanism.

To gain further insights into potential changes of the interfacial charge transfer processes electrical impedance measurements were performed on LTON_cocats and LTON_bare photoanodes after different durations of chronoamperometry.

The stability of LTON was further investigated by performing chronoamperometry of LTON_cocats in the presence of a sacrificial agent. Contrary to the chronoamperometries in standard electrolyte, a stable current density of more than 2 mA cm^−2^ was observed when the electrolyte was saturated with Na_2_SO_3_ as sacrificial agent (Fig. S27). The function of sacrificial agents is to immediately quench all charges reaching the surface.^73^ This reduces charge recombination, surface-related charge accumulation and/or degradation. The observation of a stable current density upon the addition of a sacrificial agent indicated that the bulk charge separation of the LTON remained unchanged during chronoamperometry.^73^ To investigate the charge transfer resistance at the photoanode/electrolyte interface, electrical impedance spectroscopy was performed on LTON_bare and LTON_cocats photoanodes. In the obtained Nyquist plots the charge transfer resistance is represented by the diameter of the semicircle with larger diameters indicating larger charge transfer resistances and thus less efficient photoanodes.^74–76^ The LTON_bare photoanodes exhibit consistently high charge transfer resistance, which explains their lower performance (Fig. S28). In contrast, the LTON_cocats photoanodes show in general lower charge transfer resistances which increase over the course of chronoamperometry measurements, indicating progressive degradation at the electrode/electrolyte interface. In combination with the previous observation that the bulk characteristics of LTON, such as optical properties and charge separation, did not change significantly, this a strong indication that the degradation of LTON_cocats photoanodes is surface related.

**Fig. 5:**
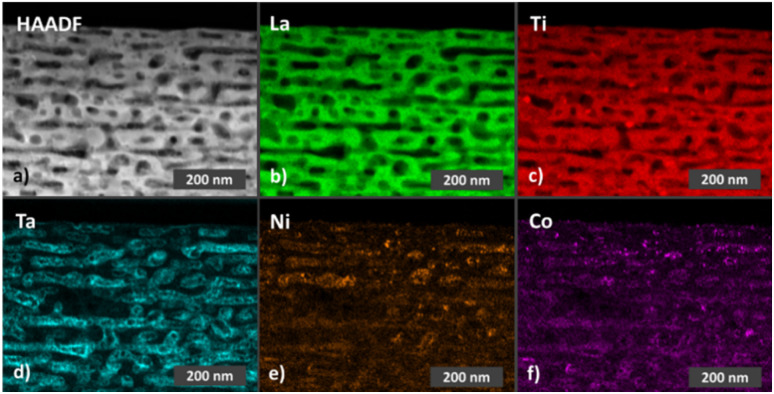
(a) HAADF STEM image of a lamella extracted from a LTON_cocats photoanode after 7 h chronoamperometry under illumination. STEM EDX intensity maps of the same lamella for (b) lanthanum, (c) titanium, (d) tantalum, (e) nickel and (f) cobalt.

In the next step we conducted structural, morphological and compositional characterization on materials and electrodes after 7 h operation under standard conditions (25 °C, AM 1.5G and 1.23 V *vs.* RHE) to identify potential degradation inflicted changes. We focused first on LTON in LTON_bare and LTON_cocats photoanodes. The XRD patterns of LTON photoanodes after stability testing did not show any change with respect to the XRD patterns of pristine LTON photoanodes (Fig. S5), suggesting that the crystal structure of the LTON remained intact. Also, the overall morphology of the LTON particles, regarding their size, shape and porosity, was found to be comparable to pristine LTON_cocats photoanodes according to SEM (Fig. 2b) and HAADF image analysis (Fig. 2c and 5a). The STEM-EDX maps of La and Ti confirmed that there have been no significant morphological changes to the LTON particles on the 10–100 nm scale (Fig. 5b and c). Since these findings were in agreement with our optical and electrochemical results, we conclude that bulk LTON was not affected by the 7 h chronoamperometries.

**Fig. 6:**
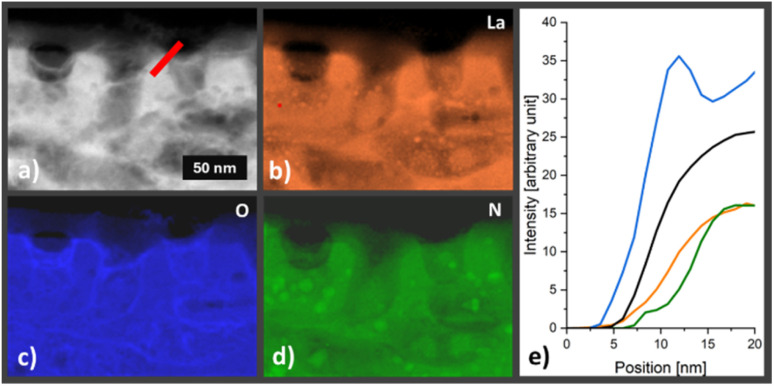
(a) ADF STEM image of a lamella extracted from a LTON_cocats photoanode after 7 h OER. EELS elemental maps of the (b) La–M edge (c) O–K edge and (d) the N–K edge of the same area. The bright green areas indicate closed pores containing N_2_. (e) Line profile of the integrated La (orange), O (blue) Ti (black) and N (green) edge intensities (red line in a).

In order to investigate potential changes of the LTON surface, we carried out XPS on LTON_bare photoanodes. We found that the nitride content at the surface of a LTON_bare photoanode was decreased by around 10% after 7 h chronoamperometry (Table S8 and Fig. S29). To localize the nitride deficiency, a TEM lamella was extracted from an LTON_cocats photoanode after OER (Fig. S30). Using electron energy loss spectroscopy (EELS), the outlines of the LTON particles were determined by the La M-edge intensity map (Fig. 6b). Comparing to the O and N K-edge maps, increased O edge intensities were observed in the outermost 2–5 nm of the LTON particle, while the N-edge intensities were decreased in the same area (Fig. 6c and d). This is visualized in a more quantitative manner in the line scan shown in Fig. 6e indicating that no N was detectable at the particle edge. Investigating the particle surfaces by HREM, we found crystalline order up to the particle surface in both pristine LTON and LTON after OER (see Fig. S31). However, the near surface layers showed a changed crystal structure in LTON after OER. Hence XPS and EELS measurements clearly indicated that a thin crystalline layer containing mainly La, Ti and O was formed at the surface during OER. Experimental proof for surface oxidation was reported for a similar material, *i.e.* Ta_3_N_5_.^32,34^ In this case, the oxide layers were up to 50 nm thick and amorphous. Consequently, surface passivation was assumed to be the main reason for the observed performance decrease of photoanodes fabricated from these nitrides. Although in our case the surface oxidation layer is significantly thinner and crystalline, we cannot exclude that it contributed to the photoactivity decrease of the LTON based photoanodes.

In the next step, we focused on degradation related to the cocatalyst system. By SEM we observed the formation of small particles (size < 50 nm) on top of the LTON_cocats photoanodes after stability testing (Fig. 2b). When analysing them by STEM-EDX, we found that Ta was still homogeneously distributed over the whole particle (Fig. 2f and 5d). Also, Ni and Co were present, but their concentrations were notably higher in specific areas of the LTON_cocats photoanode after stability testing relative to the pristine one (Fig. 2g, h and 5e, f). This observation is confirmed by XPS analysis, where an increase of both Ni and Co on the surface of a LTON_cocats photoanode was observed after OER (Fig. S6, S32 and S33). This increase is most likely caused by leaching of the cocatalyst from the open pores of the LTON particles. In combination, this is a strong indication for the formation of larger agglomerates of NiO_*x*_ and CoO_*x*_ nanoparticles on the surface of the LTON particles during chronoamperometry, hence suggesting that the cocatalyst system could rearrange under OER conditions.

To investigate potential dissolution of cations from LTON_bare and LTON_cocats photoanodes during the 7 h chronoamperometries, ICP-MS was performed (Table 3). For both types of photoanodes the concentration of La was below the detection limit of 0.1 µm L^−1^ in the electrolyte samples and the blanks (fresh electrolyte, 0.1 M Na_2_SO_4_ adjusted to pH 13.4 with added NaOH).

**Table 3:** Cation concentration in the electrolyte before and after a 7 h chronoamperometry of LTON_bare and LTON_cocats photoanodes under illumination measured with ICP-MS and the corresponding dissolution rates calculated using eqn (4)

Cation	Blank [µg L^−1^]	After 7 h chronoamperometry [µg L^−1^]	Difference [µg L^−1^]	Dissolution rate [pmol cm^−2^ s^−1^]
La_bare	<0.1	<0.1	<0.1	<0.001
Ti_bare	1.34	25.37	24.03	0.390
La_cocats	<0.1	<0.1	<0.1	<0.001
Ti_cocats	0.87	13.51	12.64	0.205
Ta_cocats	1.37	21.65	20.28	0.089
Co_cocats	<0.1	2.7	2.7	0.036

For Ti dissolution rates of 0.39 pmol cm^−2^ s^−1^ and 0.205 pmol cm^−2^ s^−1^ were observed for LTON_bare and LTON_cocats photoanodes. In the absence of La dissolution, we attribute the increase of the Ti concentrations to the dissolution of the TiO_2_ necking layer in agreement with previous measurements showing the stability of bulk LTON. The higher Ti dissolution rate observed for the LTON_bare photoanode is presumably caused by faster dissolution of the TiO_2_ without the presence of the other cocatalysts. For the LTON_cocats photoanodes dissolution rates of 0.089 pmol cm^−2^ s^−1^ and 0.036 pmol cm^−2^ s^−1^ were observed for the Ta and Co, indicating the dissolution of the Ta_2_O_5_ layer and the CoO_*x*_ cocatalyst nanoparticles during chronoamperometry. Thus, for the LTON_bare and LTON_cocats photoanodes, the different additional layers were affected by dissolution, rather than the semiconducting material itself, as is the case for oxides such as BiVO_4_.^25,28^ Since the application of cocatalysts (especially CoO_*x*_) significantly boosts the performance of LTON based photoanodes, their dissolution is expected to contribute to the observed performance decrease. This assumption is supported by the fact that LTON_cocats photoanodes showed a notably lower stability than LTON_bare photoanodes, although effects such as corrosion driven by charge accumulation are expected to be stronger without cocatalyst. Combining ICP-MS results with SEM observations showing larger particles on the surface and the XPS analysis showing an increased concentration of cocatalyst on the particle surface after chronoamperometry, cocatalyst dissolution and redeposition seems to be the most probable process. We propose that larger cocatalyst particles resulted in a smaller number of available surface reaction sites and in consequence the reaction rate and the measured current density decreased. A similar process has been suggested in literature for MnO_2_ and NiO_*x*_ cocatalysts on TaON based on SEM investigations, where larger cocatalyst particles were found after chronoamperometry.^29,30^ Thus, we propose that two surface related effects contributed to degradation in LTON based photoanodes: First, LTON is oxidized at the surface forming a 2–5 nm thick crystalline oxygen rich layer, decreasing the charge transfer efficiency at the electrode–electrolyte interface. For LTON_cocats photoanodes, a second effect is important: It is cocatalysts dissolution and redeposition, leading to decreased activity over time.

## 4 Conclusions

The degradation of oxynitride based photoanodes was investigated by performing chronoamperometries at 1.23 V *vs.* RHE with and without illumination of LTON electrodes. A detailed analysis of the experimental results revealed that the shape of the current decay was described best by a fit to a sum of two exponential decay terms. The same observation was made when fitting chronoamperometries reported in the literature for related oxynitride-based photoanodes. These results indicated that two processes are responsible for the decay of the current densities observed in oxynitride-based photoanodes. We attributed the first exponential decay term to the charging of capacitive layers or interfaces associated with the photoanode/electrolyte or the photoanode/cocatalyst/electrolyte interfaces, while the second term describes the decay of the photogenerated current with time, *i.e.*, the degradation of the photoanode. Consequently, the fitting parameters *I*_0,pc_ and *τ*_0,pc_ contain information about the photocurrent density at *t* = 0 without capacitive effects and about stability, respectively. These findings allow the calculation of an already established metric, *i.e.* current retention, based on a more precise value for the maximal current density *j*_max_. In addition, the time constant *τ*_0,pc_ was proposed as an alternative figure of merit for describing the stability of the photoanode, whose validity was demonstrated by showing its correlation to established metrics such as current retention. This metric provides the advantage that it is independent of experimental parameters such as the measurement duration or ambiguous choices such as determining the maximum photocurrent. Faraday efficiency measurements and materials characterization after stability testing revealed that the observed degradation of the LTON based photoanodes is surface-related, while bulk LTON itself is rather stable under standard operational conditions (1.23 V *vs.* RHE, AM1.5 G illumination, pH 13.4). LTON formed a 2–5 nm thick oxide layer at the surface in addition to necking and cocatalyst (*i.e.* Ti, Ta and Co) dissolution and redeposition. Since the degradation of the LTON_cocats photoanodes is mainly driven by the dissolution of the cocatalysts, future efforts should focus on increasing their chemical stability. Potential strategies should include in depth (*operando*) surface analysis to elucidate the dissolution/redeposition mechanisms of cocatalyst, followed by the development of more stable cocatalysts. Another important field is the detailed investigation of degradation as a function of operational parameters such as bias, current density and illumination with the aim of identifying suitable device operating points.

In addition, we demonstrate the importance of conducting a detailed analysis of current density evolution over time for the specific case of LTON-based photoanodes. This is necessary in order to identify potential parasitic processes, such as capacitive currents, and mitigate their effect when estimating experimental current densities at *t* = 0, which could distort efficiency assessments based on photocurrent densities.

## Conflicts of interest

There are no conflicts to declare.

## Supplementary Material

TA-014-D5TA06368J-s001

TA-014-D5TA06368J-s002

## Data Availability

The data supporting this article have been included as part of the supplementary information (SI). Supplementary information: material and photoanode characterization, on photoelectrochemical characterization, on fitting results of chronoamperometries and on degradation studies are provided. See DOI: https://doi.org/10.1039/d5ta06368j.
