# Supplementary material for: Degradation of oxynitride based photoanodes
Source: J Mater Chem A Mater. 2025 Dec 15;14(6):3605–19. doi: 10.1039/d5ta06368j (PMC12703832; doi:10.1039/d5ta06368j)
Supplement: TA-014-D5TA06368J-s002 [file TA-014-D5TA06368J-s002.pdf]

Supplementary Information (SI) for Journal of Materials Chemistry A.
This journal is © The Royal Society of Chemistry 2025

Supporting Information

Degradation of Oxynitride Based Photoanodes

*Julian Hörndl, Jakub Zalesak, Franky E. Bedoya-Lora, Sophia Haussener, and Simone Pokrant**

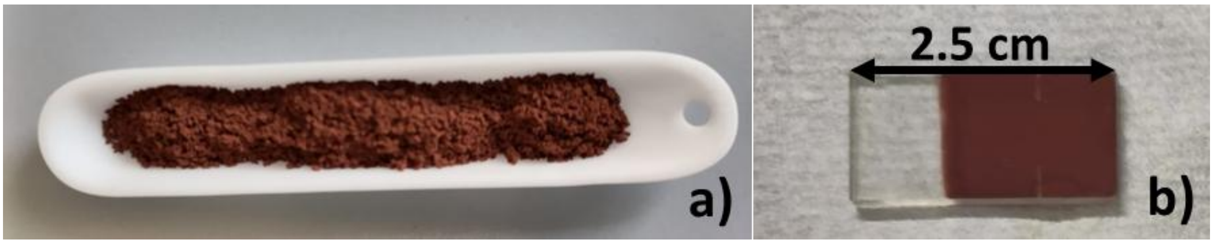

Figure S1: a) LTON powder and b) LTON based photoanode.

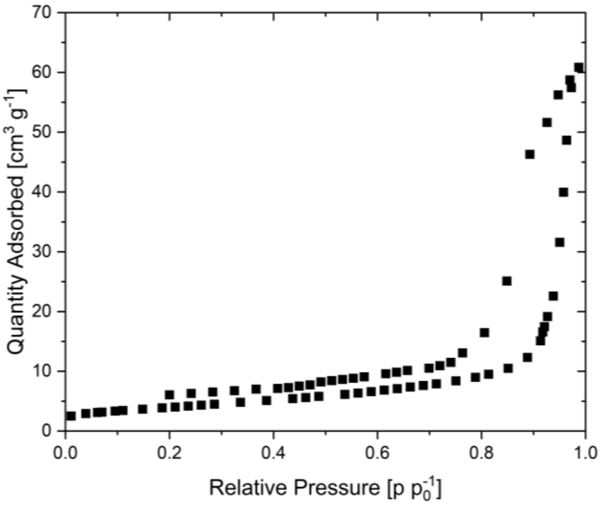

Figure S2: N_2 -Sorption measurement of LTON Powder, carried out with a Micromeritics ASAP 2040 Surface Area and Porosity Analyser. For the measurements liquid nitrogen with a temperature of 77 K was used. Before the measurements the powder was degassed for 2 h at 150 °C. The obtained BET Surface area is 14.2 g m^{-2}.

Table S1:Direct band gaps of LTON powder, LTON_bare and LTON_cocats photoanodes before and after stability testing.

Sample	Direct band Gap [eV]
LTON - Powder	2.11 ± 0.05
LTON_bare photoanode pristine	2.11 ± 0.05
LTON_bare photoanode after stability testing	2.11 ± 0.05
LTON_cocats photoanode pristine	2.08 ± 0.05
LTON_cocats photoanode after stabiltiy testing	2.09 ± 0.05

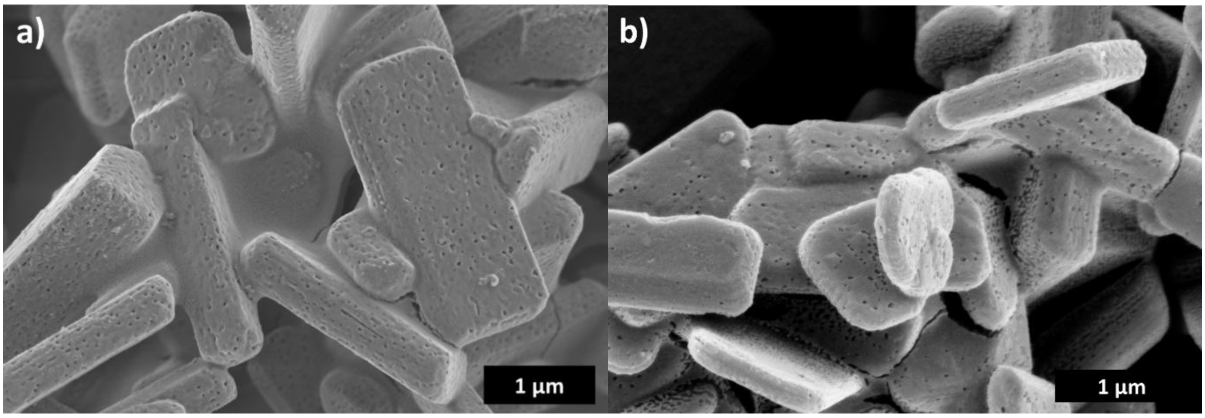

Figure S3: SEM images of (a) pristine LTON_cocats photoanodes and (b) pristine LTON_bare photoanodes (scale bars: 1 µm). The matrix-like structure between the LTON particles is attributed to TiO2 necking.

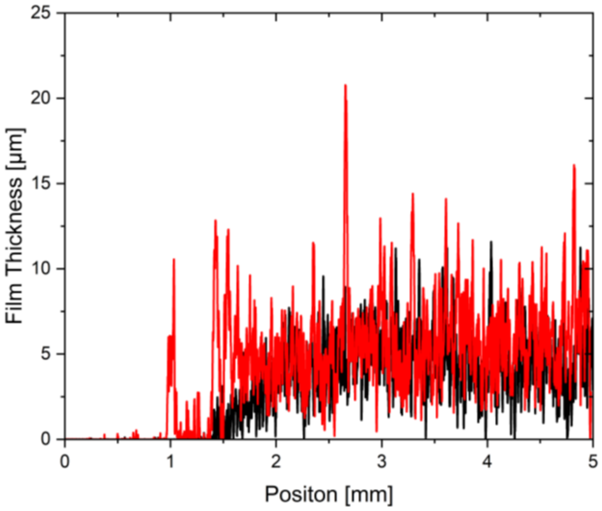

Figure S4: Thickness of the LTON particle film on top of the FTO substrate for two representative photoanodes. For each photoanode three profilometry measurements were performed and the average values were calculated at each position. Then the electrode thickness was obtained by averaging the thickness between the positions 2 mm and 5 mm.

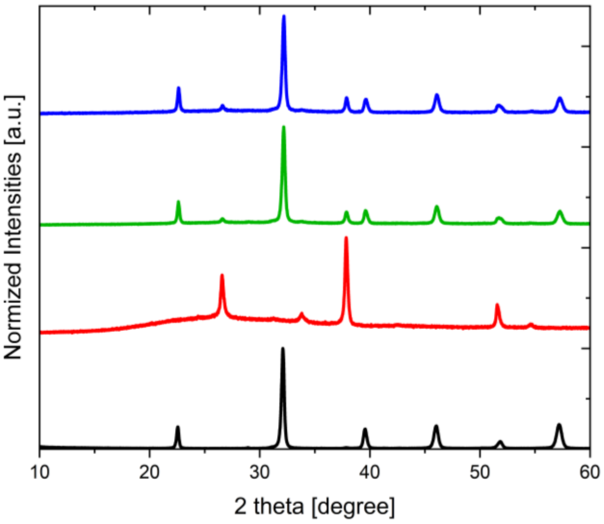

Figure S5: XRD patterns of LTON powder (black), a bare substrate (red), a pristine LTON based photoanode (green) and a LTON based photoanode after stability testing (blue).

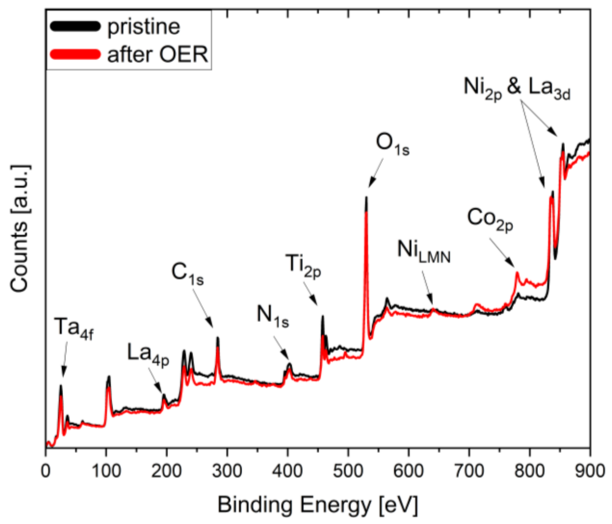

Figure S6: XPS spectra of a pristion LTON_cocats photoanode and a LTON_cocats photoaode after 7 h OER.

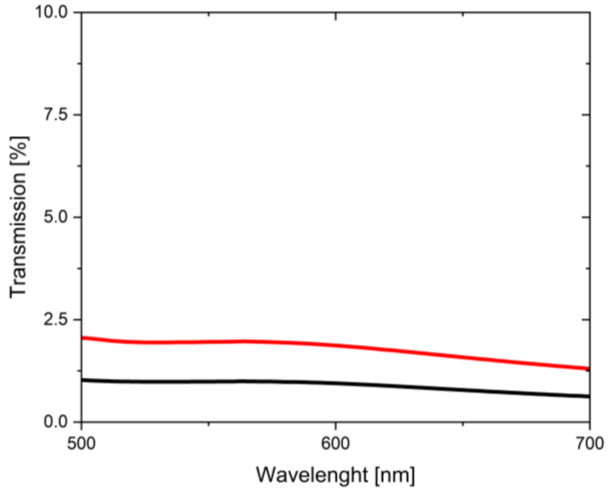

Figure S7: UV-VIS transmission spectra of LTON_bare (red) and LTON_cocats (black) photoanodes.

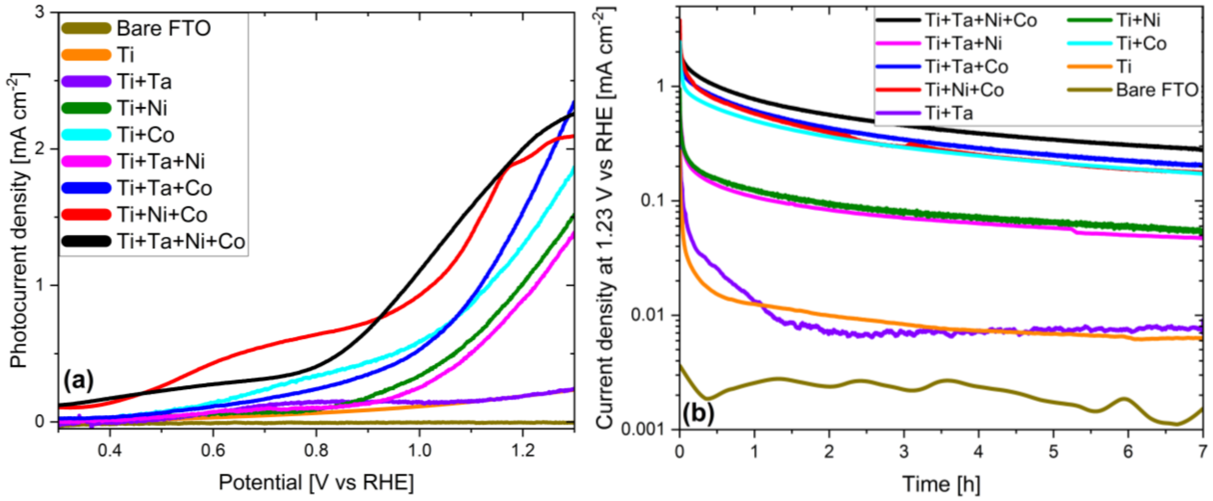

Figure S8: a) Photocurrent densities and b) chronoamperometries of LTON based photoanodes with different combinations of overlayers and of bare FTO substrates.

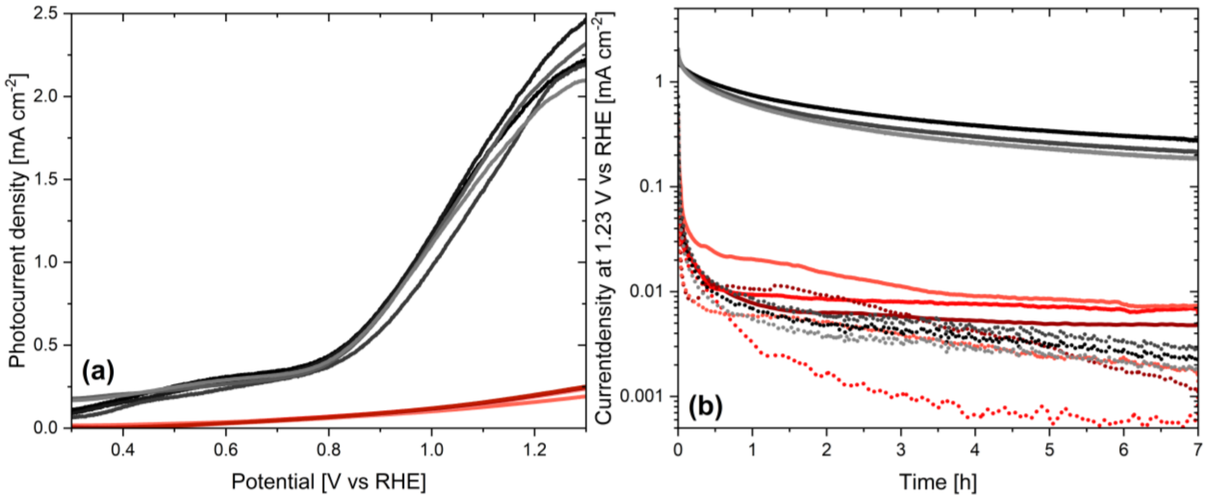

Figure S9: a) Photo current densities of five LTON_bare (red) and five LTON_cocats (black) photoanodes as a function of the applied bias potential. b) Current densities at 1.23 V vs RHE of three LTON_bare (red) and three LTON_cocats (black) photoanodes with illumination. The corresponding chronoamperometries without illumination are shown as dotted line.

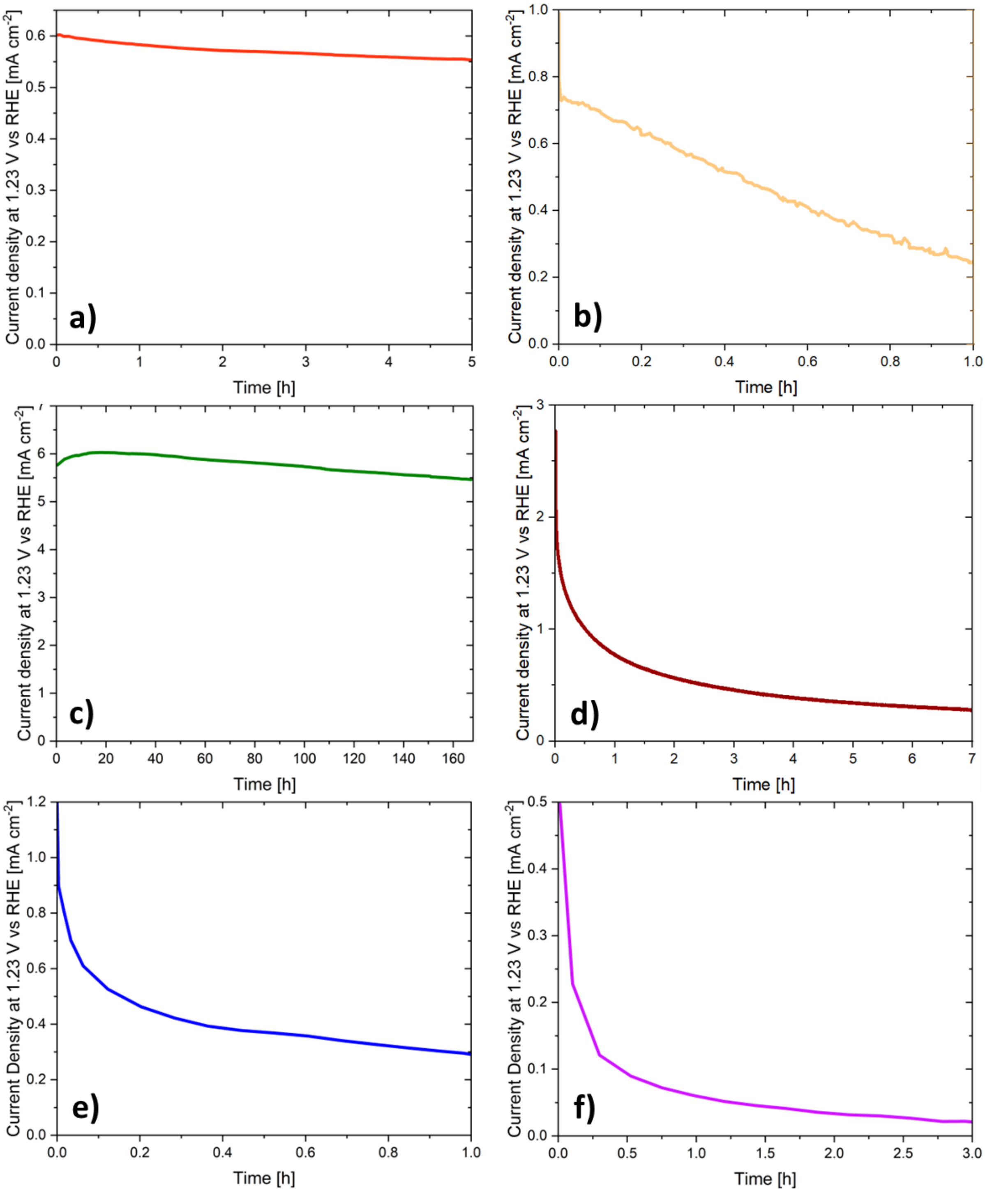

Figure S10: Current densities at 1.23 V vs RHE under 1 sun illumination obtained with a a) Fe_2O_3, Reproduced under terms of the CC-BY license.[1] Copyright 2025, V. Benavente Llorente, K.J. Jenewein, M. Bierling, A. Körner, A. Hutzler, A. Kormányos, S. Cherevko, published by American Chemical Society b) $BiVO_4$ Reproduced under terms of the CC-BY license.[2]Copyright 2025, F. M. Toma, J.K. Jason, V. Kunzelman, M.T. McDowell, J. Yu, D.M. Larson, N.J. Borys, C. Abelyan, J.W. Beeman, K. M. Yu, J. Yang, C. Le, M. R. Shaner, J. Spurgeon, F.A. Houle, K.A. Persson, I. D. Sharp, published by American Chemical Society. c) $BiVO_4$ + FeOOH/NiOOH, Reproduced under terms of the CC-BY license.[3] Copyright 2025, Z. Zhu, M. Daboczi, M. Chen, Y. Xuan, X. Liu, S. Eslava, published by American Chemical Society, d) $LaTiO_2N$ + NiO_x/CoO_x (this work) based photoanode, e) $SrTaO_2N/CoP_i$ reproduced with permission.[4] Copyright 2025, Wiley and f) Ta_3N_5, reproduced with permission.[5] Copyright 2025, Elsevier.

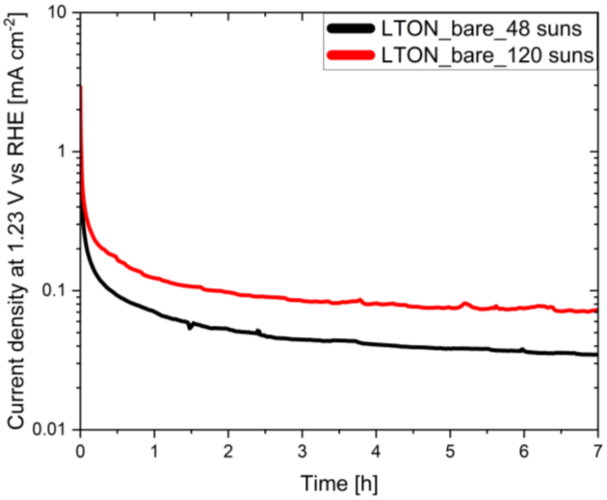

Figure S11: Chronoamperometries of LTON_bare photoanodes under illumination intensities of 48 and 120 suns.

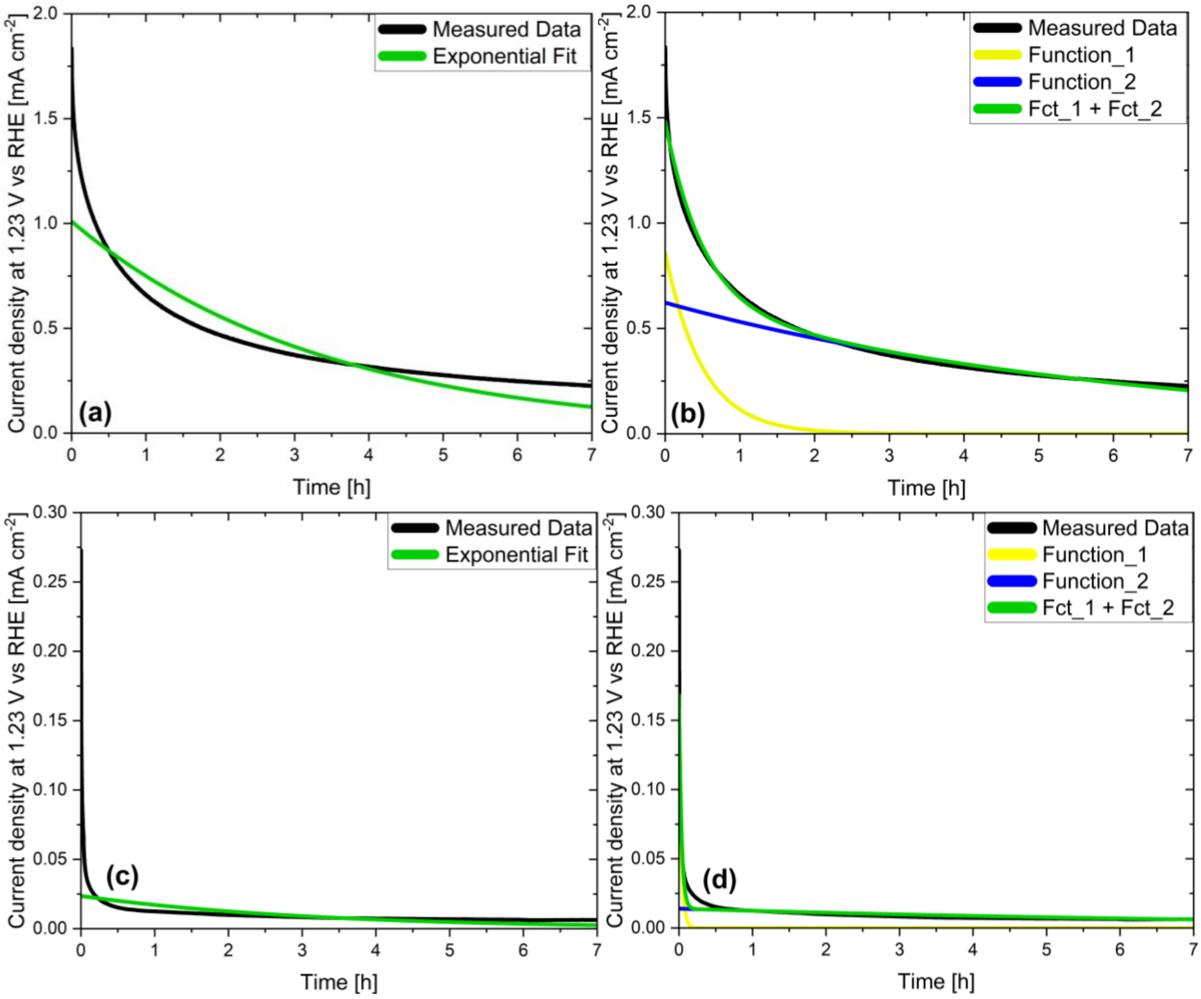

Figure S12: Fitting of the chronoamperometry obtained from LTON_cocats (a) and (b) and LTON_bare (c) and (d) photoanodes under 1 sun illumination with a decay function consisting of (a) and (c) one or (b) and (d) two exponential terms (Y-Axis with a linear scale).

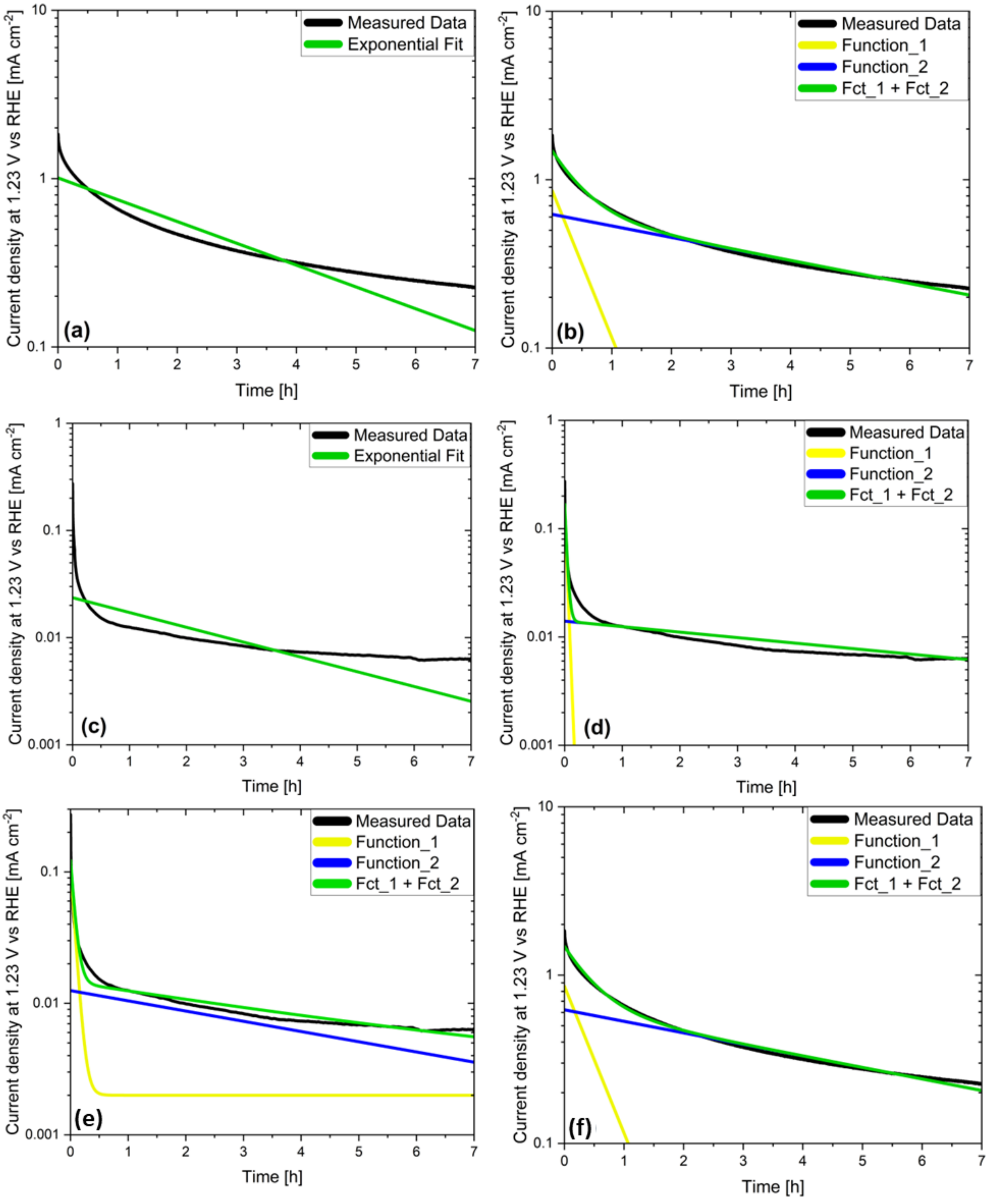

Figure S13: Fitting of the chronoamperometry obtained from LTON_cocats (a), (b) and (e) and LTON_bare (c), (d) and (f) photoanodes under 1 sun illumination with a decay function consisting of (a) and (c) one or (b) and (d) two exponential terms (Y-Axis with an exponential scale). For (e) and (f) the two exponential terms and a constant current density of 0.002 mA cm^{-2} were used to account for the dark current density. Comparing to (b) and (d) shows that the fit is sensitive to the dark current density in the case of extremely low current densities as observed for LTON_bare. Since even for LTON_bare reasonable adjusted R^2 values were obtained, a fitting model without dark current was used.

Table S2: Fitting Parameters for fits with one exponential Function

Photocatalyst	Cocatalyst	τ [min]	I_0 [mA cm^{-2}]	R^2	Reference
$LaTiO_2N$	Co_3O_4	2.19	2.89	0.534	Feng *et al.* [6]
$LaTiO_2N$	Co_3O_4	91.3	1.38	0.758	Mao *et al.* [7]
$Na_{0.1}La_{0.9}TiO_{2.2}N_{0.8}$	Co_3O_4	42.8	0.367	0.549	Mao *et al.* [7]
$SrTaO_2N$	--	13.6	0.14	0.721	Zhong *et al.* [4]
$SrTaO_2N$	CoP_i	35.3	0.891	0.756	Zhong *et al.* [4]
$BaNbO_2N$	FeO_x | $Co(OH)_x$	53.1	3.29	0.828	Seo *et al.* [8]
TaON	--	0.022	1.83	0.986	Higashi *et al.* [9]
TaON	CoO_x	176	0.929	0.117	Higashi *et al.* [9]
TaON	IrO_x	928	0.779	0.772	Higashi *et al.* [9]
TaON	CoO_x	209	0.901	0.104	Higashi *et al.* [9]
Ta_3N_5	--	13.6	0.479	0.956	He *et al.* [5]
$LaTiO_2N$	CoO_x &NiO_x	201	1.01	0.859	This work
$LaTiO_2N$	--	118	0.0255	0.335	This work
$LaTiO_2N$_dark	CoO_x &NiO_x	0.60	0.552	0.779	This work
$LaTiO_2N$_dark	--	128	0.0136	0.506	This work

Table S3: Fitting parameters for fits with two exponential functions.

Photocatalyst	Cocatalyst	$τ_{cp}$ [min]	$I_{0,cp}$ [mA cm^{-2}]	$τ_{pc}$ [min]	$I_{0,pc}$ [mA cm^{-2}]	R^2	Reference
$LaTiO_2N$	Co_3O_4	0.90	2.30	235	0.851	0.987	Feng *et al.* [6]
$LaTiO_2N$	Co_3O_4	3.67	0.84	192	0.947	0.987	Mao *et al.* [7]
$Na_{0.1}La_{0.9}TiO_{2.2}N_{0.8}$	Co_3O_4	0.26	0.48	112	0.193	0.995	Mao *et al.* [7]
$SrTaO_2N$	--	1.83	0.14	62.9	0.051	0.982	Zhong *et al.* [4]
$SrTaO_2N$	CoP_i	1.47	0.55	86.5	0.547	0.973	Zhong *et al.* [4]
$BaNbO_2N$	FeO_x|$Co(OH)_x$	2.43	1.54	86.62	2.570	0.991	Seo *et al.* [8]
TaON	--	0.85	0.27	37.7	0.210	0.999	Higashi *et al.* [9]
TaON	CoO_x	0.42	1.17	1224	0.771	1	Higashi *et al.* [9]
TaON	IrO_x	2.87	0.25	130	0.454	0.988	Higashi *et al.* [9]
TaON	CoO_x	0.29	1.05	1538	0.768	1	Higashi *et al.* [9]
Ta_3N_5	--	4.59	0.44	89.2	0.127	0.999	He *et al.* [5]
$LaTiO_2N$	CoO_x &NiO_x	30.3	0.86	387	0.609	0.994	This work
$LaTiO_2N$	--	4.02	0.11	335	0.013	0.935	This work
$LaTiO_2N$_dark	CoO_x &NiO_x	0.60	0.56	130	0.016	0.944	This work
$LaTiO_2N$_dark	--	1.14	0.09	172	0.011	0.963	This work

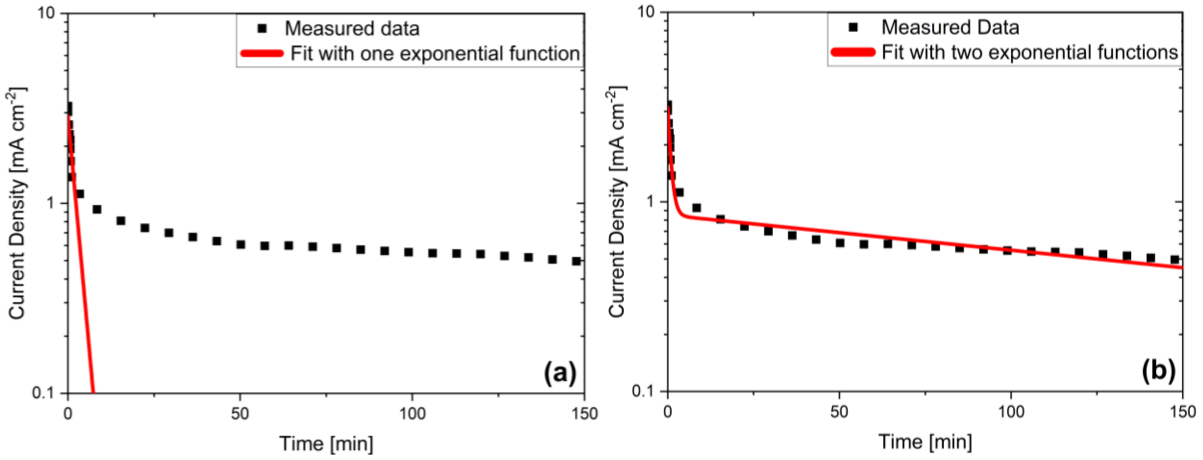

Figure S14: Chronoamperometry of LTON/Co_3O_4 measured by Feng et al. fitted with a) one and b) two exponential functions, reproduced with permission.[6] Copyright 2025, Wiley.

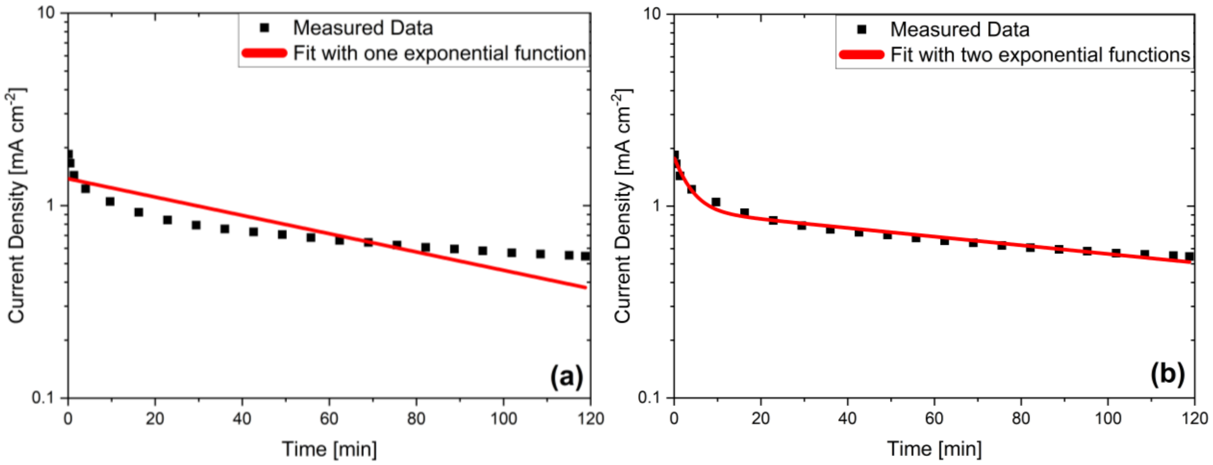

Figure S15: Chronoamperometry of $LaTiO_2N$/Co_3O_4 measured by Mao et al. fitted with a) one and b) two exponential functions, reproduced with permission.[7] Copyright 2025, Elsevier.

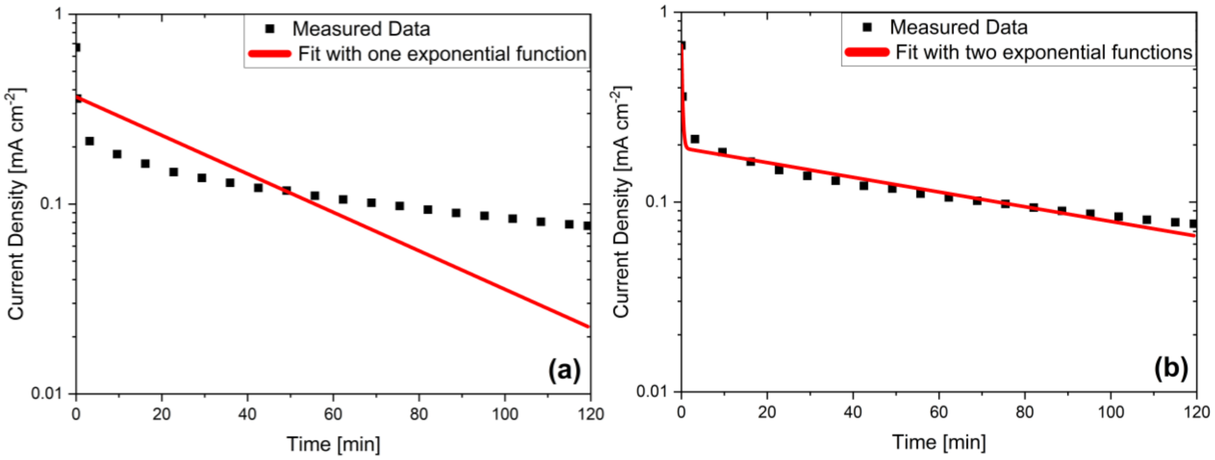

Figure S16: Chronoamperometry of $Na_{0.1}La_{0.9}TiO_{2.2}N_{0.8}$/$Co_3O_4$ measured by Mao et al. fitted with a) one and b) two exponential functions, reproduced with permission.[7] Copyright 2025, Elsevier.

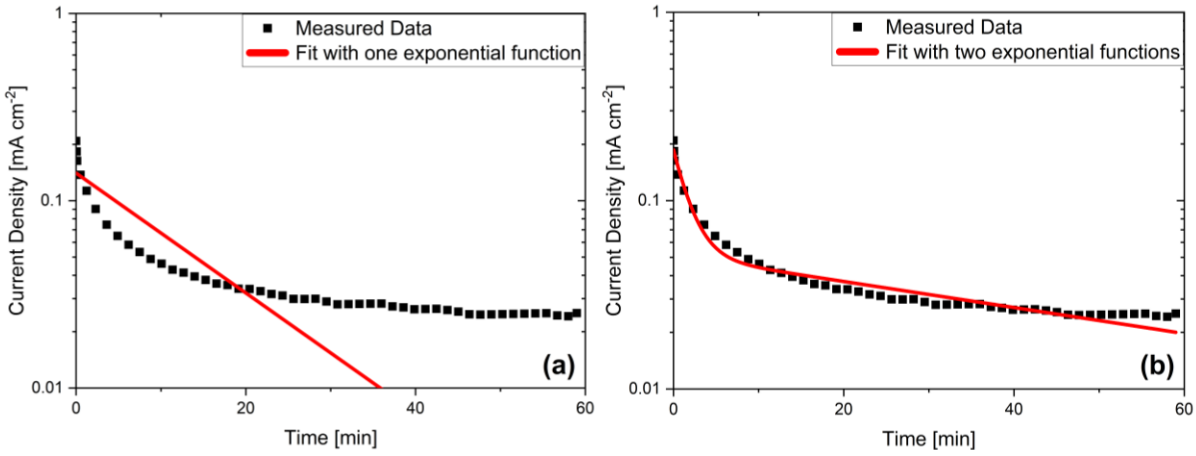

Figure S17: Chronoamperometry of $SrTaO_2N$ measured by Zhong et al. fitted with a) one and b) two exponential functions, reproduced with permission.[4] Copyright 2025, Wiley.

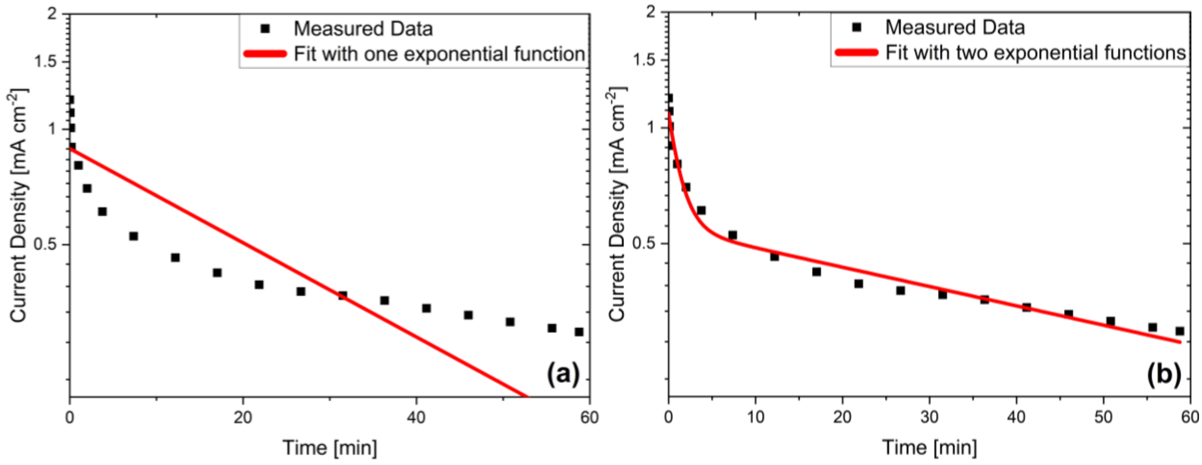

Figure S18: Chronoamperometry of $SrTaO_2N/CoP_i$ measured by Zhong et al. fitted with a) one and b) two exponential functions, reproduced with permission.[4] Copyright 2025, Wiley.

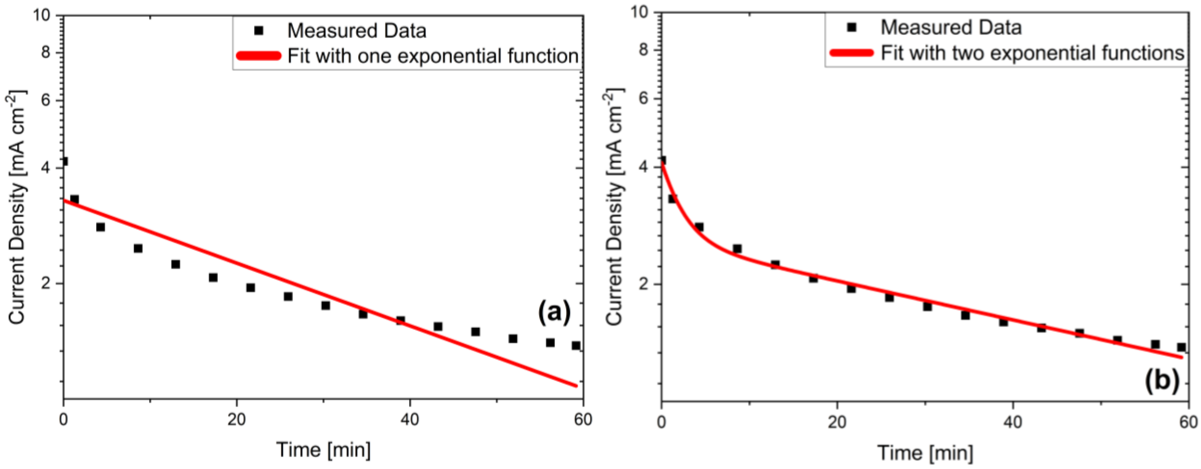

Figure S19: Chronoamperometry of $BaNbO_2N/FeO_x|Co(OH)_x$ measured by Seo et al. fitted with a) one and b) two exponential functions, reproduced with permission.[8] Copyright 2025, Wiley.

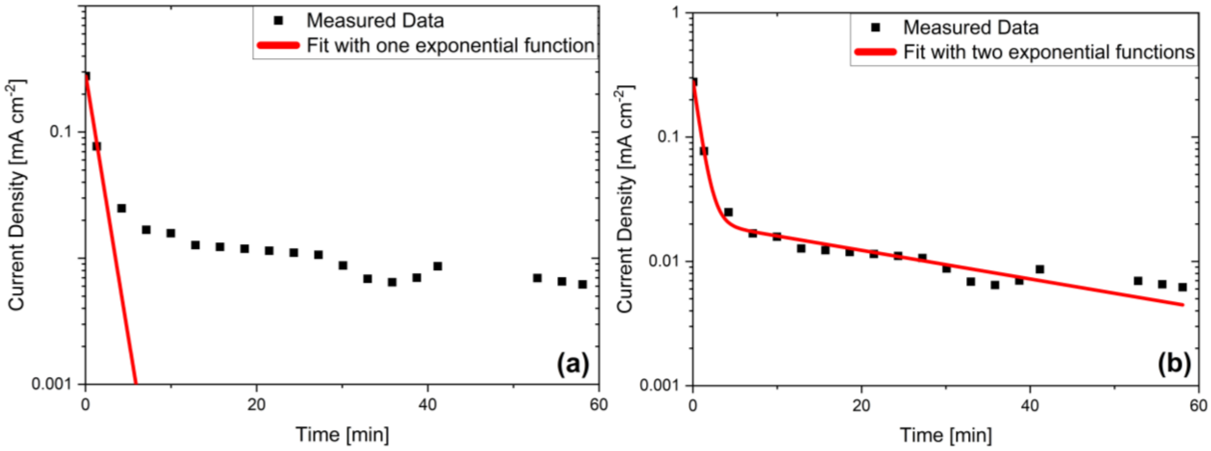

Figure S20: Chronoamperometry of TaON measured by Higashi et al. fitted with a) one and b) two exponential functions, reproduced with permission.[9] Copyright 2025, American Chemical Society.

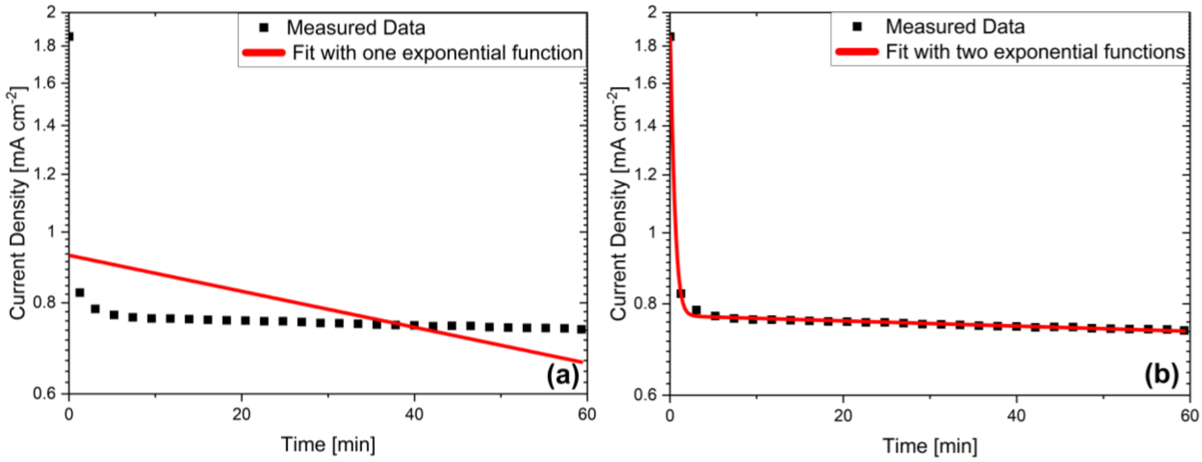

Figure S21: Chronoamperometry of $TaON/CoO_x$ measured by Higashi et al. fitted with a) one and b) two exponential functions, reproduced with permission.[9] Copyright 2025, American Chemical Society.

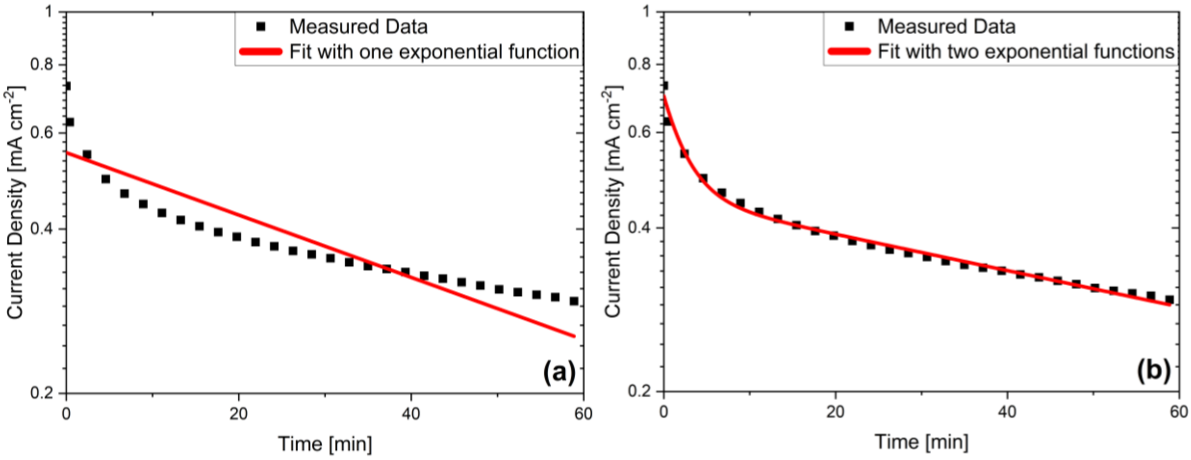

Figure S22: Chronoamperometry of TaON/IrO_x measured by Higashi et al. fitted with a) one and b) two exponential functions, reproduced with permission.[9] Copyright 2025, American Chemical Society.

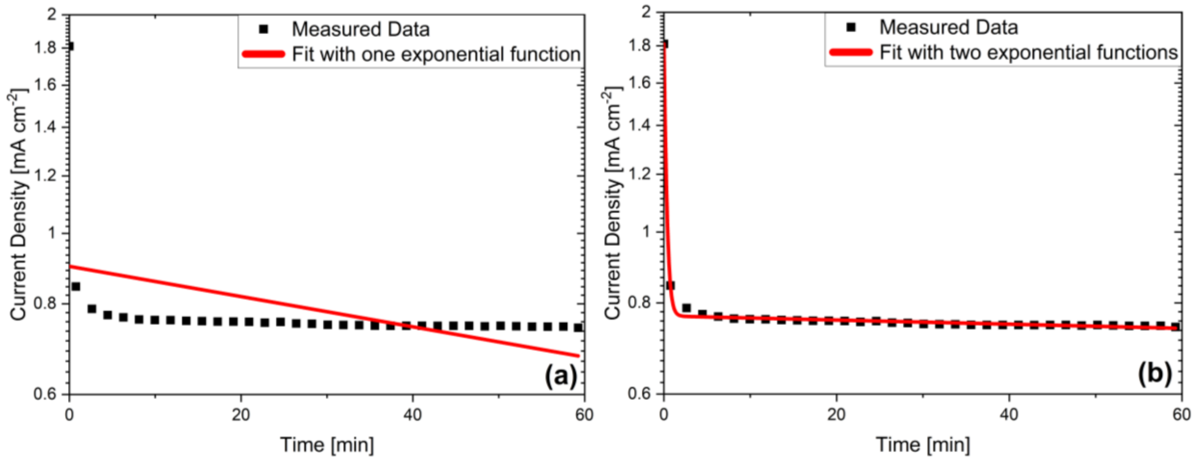

Figure S23: Chronoamperometry of TaON/CoO_x measured by Higashi et al. fitted with a) one and b) two exponential functions, reproduced with permission.[9] Copyright 2025, American Chemical Society.

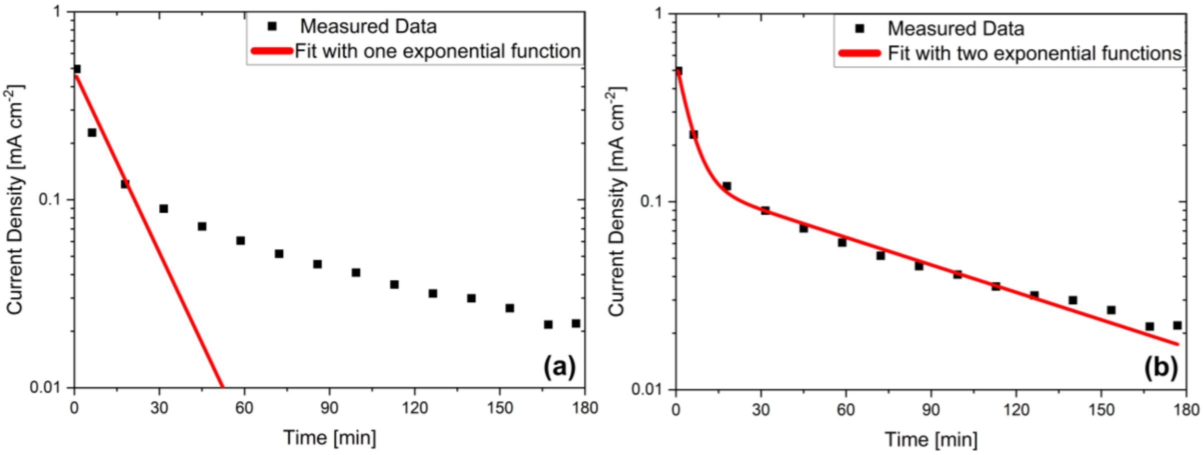

Figure S24: Chronoamperometry of Ta_3N_5 measured by He et al. fitted with a) one and b) two exponential functions, reproduced with permission.[5] Copyright 2025, Elsevier.

Table S4: Capacitive behaviour of oxynitride photoanodes: capacitive charge Q_{cp60} and the total charge Q_{tot60} after 60 minutes and the fitting parameter $I_{0,cp}$ and the measured current density at t=0.

Photocatalyst	Q_{cp60} [C]	Capacitance [mF]	Q_{tot60} [C]	Ratio Q_{cp60}/Q_{tot60} [%]	Ratio $I_{0,cp}/j_{t=0}$ [%]	Reference
$LaTiO_2N$	0.124	76.1	2.82	4.4	70.6	Feng *et al.* [6]
$LaTiO_2N$	0.186	114	3.12	6.0	45.7	Mao *et al.* [7]
$Na_{0.1}La_{0.9}TiO_{2.2}N_{0.8}$	0.007	4.3	0.54	1.4	71.5	Mao *et al.* [7]
$SrTaO_2N$	0.015	9.2	0.132	11.2	64.6	Zhong *et al.* [4]
$SrTaO_2N$	0.048	29.4	1.446	3.3	45.6	Zhong *et al.* [4]
$BaNbO_2N$	0.224	137	6.846	3.3	37.0	Seo *et al.* [8]
TaON	0.014	8.6	0.055	25.0	97.1	Higashi *et al.* [9]
TaON	0.029	17.8	2.72	1.1	63.1	Higashi *et al.* [9]
TaON	0.042	25.8	1.332	3.2	33.6	Higashi *et al.* [9]
TaON	0.018	11.0	2.706	0.68	57.8	Higashi *et al.* [9]
Ta_3N_5	0.12	7.4	0.474	25.3	87.7	He *et al.* [5]
$LaTiO_2N$_cocats	1.562	958	3.895	40.1	31.0	This work
$LaTiO_2N$_necked	0.026	16.3	0.077	34.0	40.3	This work
$LaTiO_2N$_cocats_dark	0.020	16.4	0.061	32.8	96.9	This work
$LaTiO_2N$_necked_dark	0.006	4.9	0.036	16.7	67.7	This work

The variations within the same material system can be attributed to differences in the electrode designs, including the different cocatalysts, and measurement set-ups, since parameters such as electrolyte, photoactive material, photoanode size, distance between working and counter electrode may influence the capacitance of the whole system.

Table S5: Capacitive charge and capacitance of LTON based photoanodes with different cocatalyst layers, with and without illumination.

	Under Illumination		No Illumination		
	Capacitive Charge [As]	Capacitance [mF]	Capacitive Charge [As]	Capacitance [mF]	Ratio [%]
LTON+Ti+Ta+Ni+Co	1.56	958	0.020	16.4	1.7
LTON+Ti+Ta+Ni	0.101	62.0	0.008	6.2	10
LTON+Ti+Ta+Co	1.39	858	0.050	40.1	4.7
LTON+Ti+Ni+Co	1.09	673	0.017	14.0	2.0
LTON+Ti+Ta	0.044	27.0	0.003	2.0	7.4
LTON+Ti+Ni	0.116	71.0	0.007	5.3	7.5
LTON+Ti+Co	1.06	652	0.014	10.8	1.7
LTON+Ti	0.026	16.3	0.006	4.9	41.2

Investigating this effect more closely by repeating these experiments with and without illumination after the application of each cocatalyst layer, we find that that the difference between the capacitance obtained from the measurement under illumination and the measurement in the dark increased after the addition of each cocatalyst layer. This indicates that the different cocatalysts and their interactions between them are responsible for the large increase of the capacitance of the photoanode under illumination.

Table S6: Current retentions after 60 minutes based on $j_{t=0}$ and the fitting parameter $I_{0,pc}$ as initial values.

**Photocatalyst**	**$j_{t=0}$ [mA cm^{-2}]**	**$I_{0,pc}$ [mA cm^{-2}]**	**$j_{t=60}$ [mA cm^{-2}]**	**$Ret_{t=0}$ [%]**	**$Ret_{I\_0,pc}$ [%]**	Reference
$LaTiO_2N$	3.253	0.851	0.6	18.4	70.5	Feng *et al.* [6]
$LaTiO_2N$	1.849	0.947	0.67	36.2	70.8	Mao *et al.* [7]
$Na_{0.1}La_{0.9}TiO_{2.2}N_{0.8}$	0.669	0.193	0.108	16.1	56.0	Mao *et al.* [7]
$SrTaO_2N$	0.209	0.051	0.024	11.5	47.1	Zhong *et al.* [4]
$SrTaO_2N$	1.196	0.547	0.294	24.6	53.7	Zhong *et al.* [4]
$BaNbO_2N$	4.167	2.570	1.375	33.0	53.5	Seo *et al.* [8]
TaON	0.278	0.210	0.006	2.2	2.9	Higashi *et al.* [9]
TaON	1.854	0.771	0.763	41.2	99.0	Higashi *et al.* [9]
TaON	0.732	0.454	0.292	39.9	64.3	Higashi *et al.* [9]
TaON	1.811	0.768	0.739	40.8	96.2	Higashi *et al.* [9]
Ta_3N_5	0.497	0.127	0.06	12.1	47.2	He *et al.* [5]
$LaTiO_2N$_cocats	2.772	0.609	0.775	28.0	127.3	This work
$LaTiO_2N$_necked	0.273	0.013	0.013	4.8	98.3	This work
$LaTiO_2N$_cocats_dark	0.579	0.016	0.007	1.2	43.8	This work
$LaTiO_2N$_necked_dark	0.133	0.011	0.007	5.6	63.6	This work

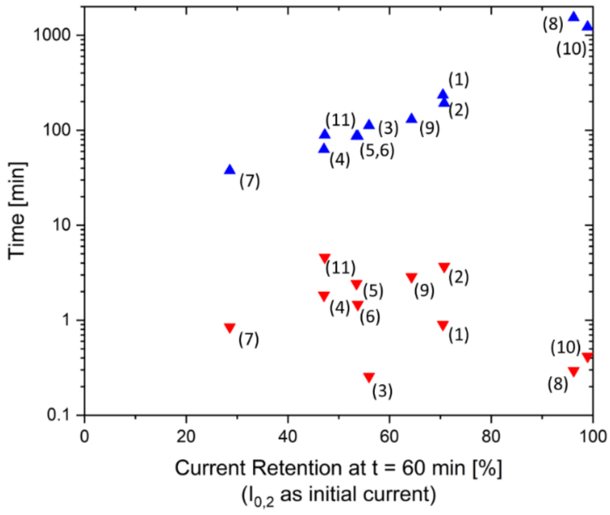

Figure S25: Time constants of the fit with two exponential terms as a function of the current retention after 60 minutes based on the fitting parameter $I_{0,2}$ as j_{max} (red triangles: τ_{cp} and blue triangles: τ_{pc}).(1) Feng *et al.*, [4] (2 & 3) Mao *et al.*,[5] (4 & 5) Zhong *et al.*,[6] (6) Seo *et al.*,[7] (7 – 10) Higashi *et al.*,[8] (11) He *et al.*.[9]

Table S7: Stability of LTON-photoanodes decorated with various cocatalyst combinations obtained by fitting 7h chronoamperometries (Figure S8b) by Equation 4

**Necking/Cocatalyst system**	**τ_{pc} [h]**	**I0,pc [mA cm.2]**	**Ret_{I0pc} [%]**
Ti	5.58	0.013	48.3
Ti+Ta	6.43	0.015	53.3
Ti+Ni	5.73	0.148	37.2
Ti+Co	5.05	0.569	30.4
Ti+Ta+Ni	5.34	0.136	34.6
Ti+Ta+Co	5.71	0.607	33.9
Ti+Ni+Co	3.92	0.723	24.0
Ti+Ta+Ni+Co	6.27	0.767	36.1

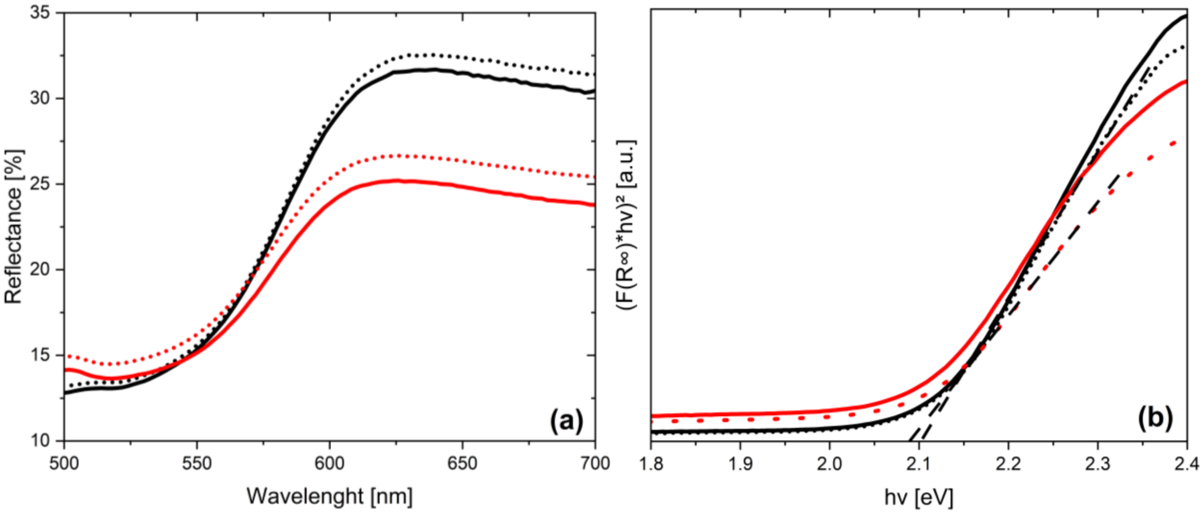

Figure S26: (a) Diffuse Reflectance spectra and (b) Tauc-Plots of the Kubelka-Munk Functions LTON_bare photoanodes (black) and LTON_cocats photoanodes (red), before (full line) and after stability testing (dotted line).

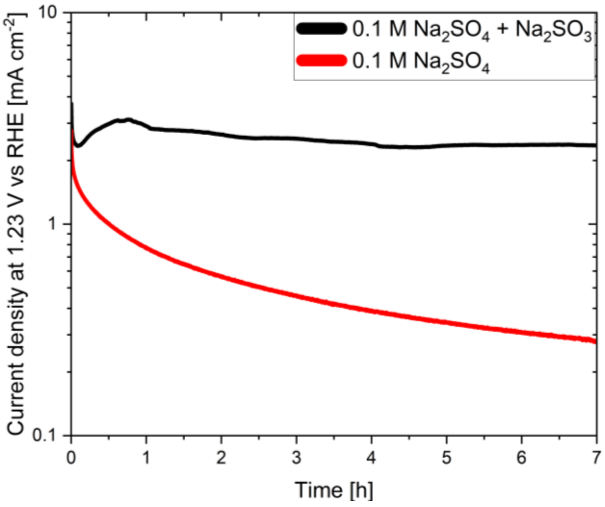

Figure S27: 7 h chronoamperometries at 1.23 V vs RHE and under 1 sun illumination of LTON_cocats photoanodes with and without sacrificial agent in the electrolyte.

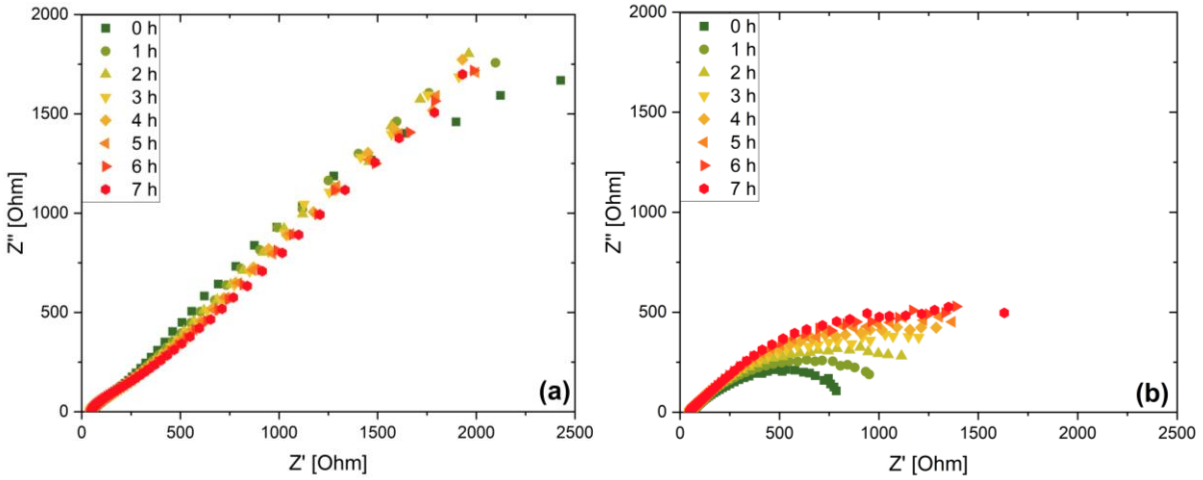

Figure S28: Electrical impedance spectroscopy performed of a) LTON_bare and b) LTON_cocats photoanodes after each hour of chronoamperometry under illumination at 1.23 V vs RHE

Table S8: Surface composition of LTON_bare photoanodes before and after 7 h chronoamperometry, labelled as after OER, measured via XPS.

**Sample**	**Ti [atom. %]**	**La [atom. %]**	**O [atom. %]**	**N [atom. %]**	**thereof N^{3-} [atom. %]**	**N^{3-} per formula unit**
LTON_bare pristine	18.39	10.77	60.87	9.94	7.52	0.69
LTON_bare after OER	16.86	8.75	67.58	6.79	5.20	0.59

Since the O and Ti signals contain contributions from the TiO_2 necking, the La content ist suited best as reference. Consequentliy the amount of N^{3-} per formula unit was derived from the ratio between the La and the N^{3-}. The additional N_2 contribution comes from the nitrogen trapped in the closed pores of LTON during thermal amonolysis.

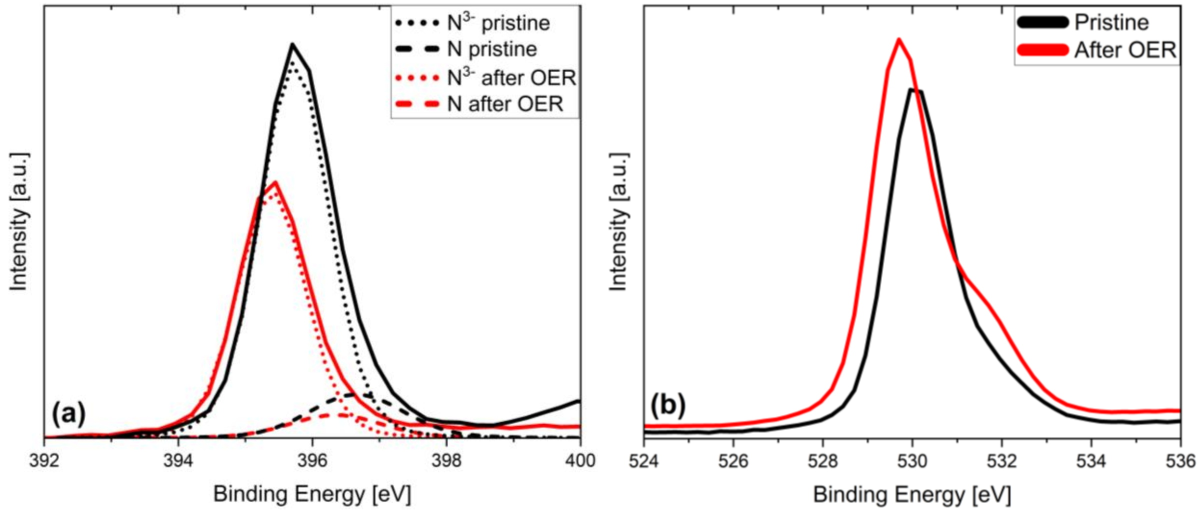

Figure S29: High resolution XPS spectra of a) N1s and b) O1s for pristine and after 7 h OER LTON_bare photoanodes

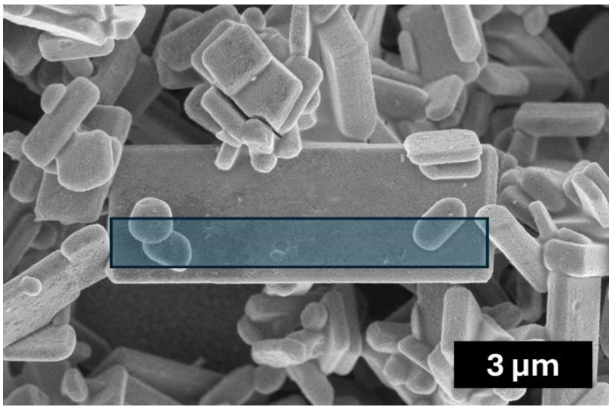

Figure S30: SEM image of a LTON_cocats photanode (top view). The blue box shows the location of FIB lamella preparation.

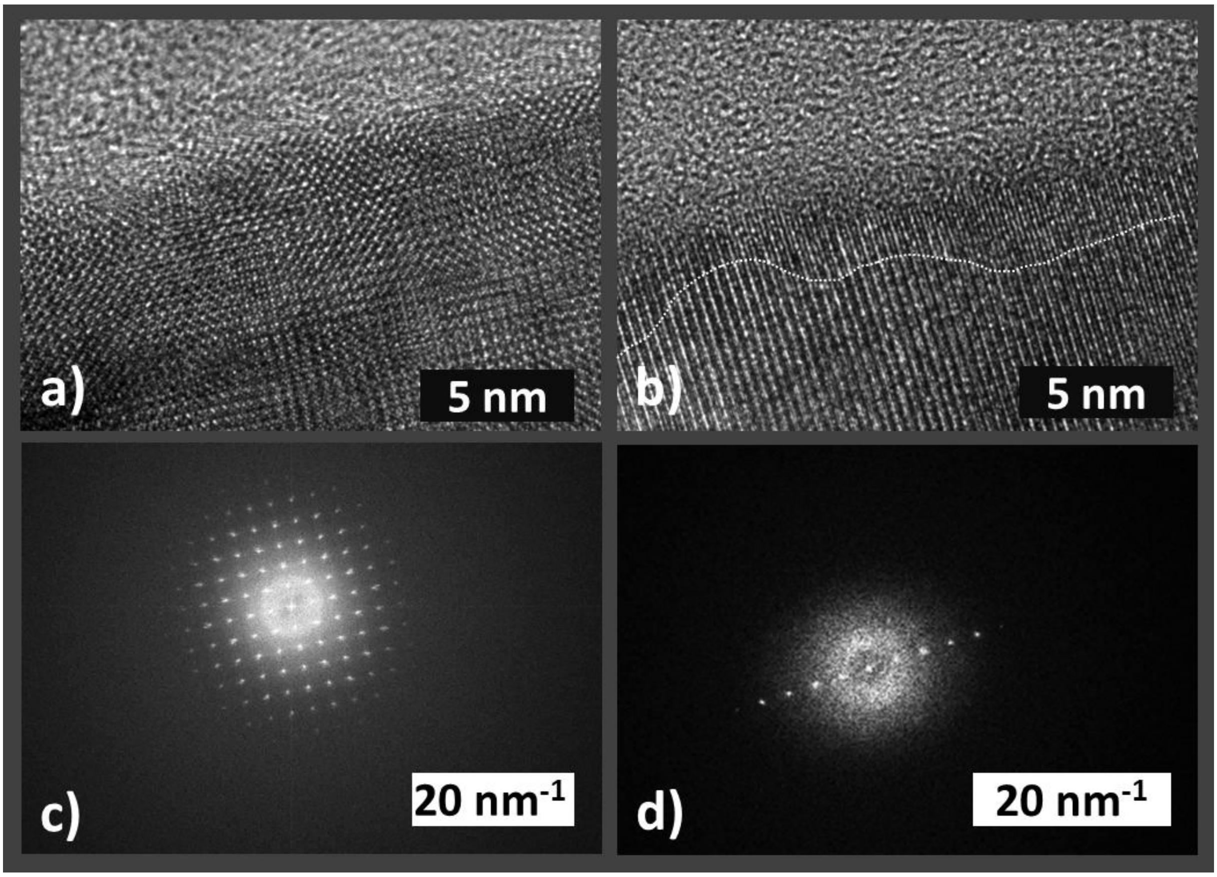

Figure S31: HREM images of LTON particle surfaces obtained from lamella extracted from a) a pristine LTON_cocats photoanode and b) after 7 h OER. c) and d) represent the corresponding Fast Fourier Transforms (FFT) to a) and b). The FFT show that the particles were not cut in the same crystal directions, therefore showing different crystalline patterns. In the pristine sample the crystal planes extend up to the particle edge. After OER the crystalline pattern is not continuous, but additional planes appear towards the particle surface. The dotted line serves as guide to the eye.

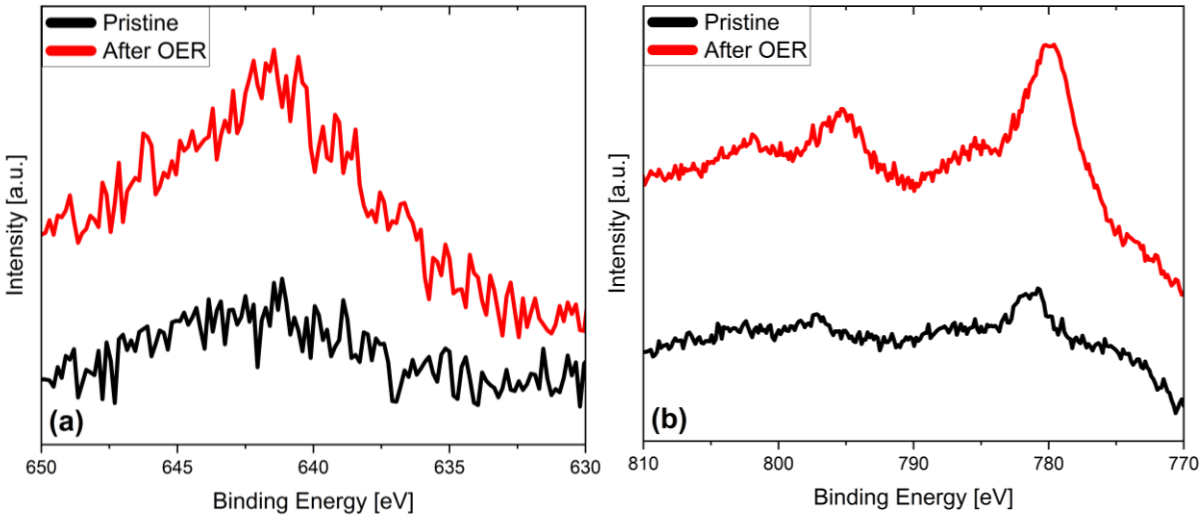

Figure S32: High resolution XPS spectra of a) NiLMM Auger and b) Co2p

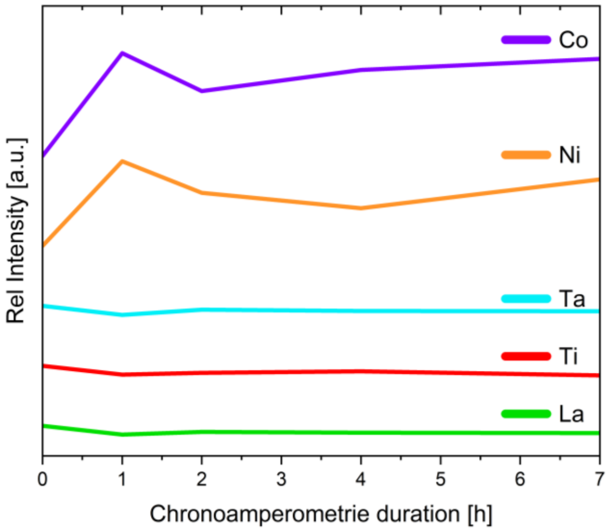

Figure S33: Evolution of the surface composition (Co, Ni, La, Ta and Ti) of LTON_cocats photoanodes as a function of the chronoamperometry duration (OER under illumination). The edge intensities are reported relative to their intensity at t=0 with arbitrary offsets for reasons of readability. The XPS spectra are shown in Figure S6.

References

1. V. Benavente Llorente, K. J. Jenewein, M. Bierling, A. Körner, A. Hutzler, A. Kormányos and S. Cherevko, *The Journal of Physical Chemistry C*, 2023, **127**, 19687-19697.
2. F. M. Toma, J. K. Cooper, V. Kunzelmann, M. T. McDowell, J. Yu, D. M. Larson, N. J. Borys, C. Abelyan, J. W. Beeman, K. M. Yu, J. Yang, L. Chen, M. R. Shaner, J. Spurgeon, F. A. Houle, K. A. Persson and I. D. Sharp, *Nature Communications*, 2016, **7**, 12012.
3. Z. Zhu, M. Daboczi, M. Chen, Y. Xuan, X. Liu and S. Eslava, *Nat Commun*, 2024, **15**, 2791.
4. Y. Zhong, Z. Li, X. Zhao, T. Fang, H. Huang, Q. Qian, X. Chang, P. Wang, S. Yan, Z. Yu and Z. Zou, *Advanced Functional Materials*, 2016, **26**, 7156-7163.
5. Y. He, James E. Thorne, Cheng H. Wu, P. Ma, C. Du, Q. Dong, J. Guo and D. Wang, *Chem*, 2016, **1**, 640-655.
6. J. Feng, W. Luo, T. Fang, H. Lv, Z. Wang, J. Gao, W. Liu, T. Yu, Z. Li and Z. Zou, *Advanced Functional Materials*, 2014, **24**, 3535-3542.
7. L. Mao, X. Cai, H. Gao, X. Diao and J. Zhang, *Nano Energy*, 2017, **39**, 172-182.
8. J. Seo, T. Hisatomi, M. Nakabayashi, N. Shibata, T. Minegishi, M. Katayama and K. Domen, *Advanced Energy Materials*, 2018, **8**, 1800094.
9. M. Higashi, K. Domen and R. Abe, *J Am Chem Soc*, 2012, **134**, 6968-6971.
